# CLRN1 Is Nonessential in the Mouse Retina but Is Required for Cochlear Hair Cell Development

**DOI:** 10.1371/journal.pgen.1000607

**Published:** 2009-08-14

**Authors:** Scott F. Geller, Karen I. Guerin, Meike Visel, Aaron Pham, Edwin S. Lee, Amiel A. Dror, Karen B. Avraham, Toshinori Hayashi, Catherine A. Ray, Thomas A. Reh, Olivia Bermingham-McDonogh, William J. Triffo, Shaowen Bao, Juha Isosomppi, Hanna Västinsalo, Eeva-Marja Sankila, John G. Flannery

**Affiliations:** 1 Helen Wills Neuroscience Institute, University of California, Berkeley, California, United States of America; 2 Vision Science, University of California, Berkeley, California, United States of America; 3 Department of Human Molecular Genetics and Biochemistry, Sackler School of Medicine, Tel Aviv University, Tel Aviv, Israel; 4 Department of Biological Structure, School of Medicine, University of Washington, Seattle, Washington, United States of America; 5 Life Sciences Division, Lawrence Berkeley National Laboratory, Berkeley, California, United States of America; 6 Folkhälsan Institute of Genetics, Biomedicum Helsinki and Department of Medical Genetics, University of Helsinki, Helsinki, Finland; 7 Helsinki University Eye Hospital, Helsinki, Finland; National Institute on Deafness and Other Communication Disorders, National Institutes of Health, United States of America

## Abstract

Mutations in the *CLRN1* gene cause Usher syndrome type 3 (USH3), a human disease characterized by progressive blindness and deafness. Clarin 1, the protein product of *CLRN1*, is a four-transmembrane protein predicted to be associated with ribbon synapses of photoreceptors and cochlear hair cells, and recently demonstrated to be associated with the cytoskeleton. To study *Clrn1*, we created a *Clrn1* knockout (KO) mouse and characterized the histological and functional consequences of *Clrn1* deletion in the retina and cochlea. *Clrn1* KO mice do not develop a retinal degeneration phenotype, but exhibit progressive loss of sensory hair cells in the cochlea and deterioration of the organ of Corti by 4 months. Hair cell stereocilia in KO animals were longer and disorganized by 4 months, and some *Clrn1* KO mice exhibited circling behavior by 5–6 months of age. *Clrn1* mRNA expression was localized in the retina using in situ hybridization (ISH), laser capture microdissection (LCM), and RT–PCR. Retinal *Clrn1* transcripts were found throughout development and adulthood by RT–PCR, although expression peaked at P7 and declined to undetectable levels in adult retina by ISH. LCM localized *Clrn1* transcripts to the retinas inner nuclear layer, and WT levels of retinal *Clrn1* expression were observed in photoreceptor-less retinas. Examination of *Clrn1* KO mice suggests that CLRN1 is unnecessary in the murine retina but essential for normal cochlear development and function. This may reflect a redundancy in the mouse retina not present in human retina. In contrast to mouse KO models of USH1 and USH2, our data indicate that *Clrn1* expression in the retina is restricted to the Müller glia. This is a novel finding, as most retinal degeneration associated proteins are expressed in photoreceptors, not in glia. If *CLRN1* expression in humans is comparable to the expression pattern observed in mice, this is the first report of an inner retinal protein that, when mutated, causes retinal degeneration.

## Author Summary

Usher syndrome (USH) is a progressive disease affecting two primary senses: vision and hearing. Often by the third decade of life, affected persons have lost the majority of their rod photoreceptors, which leads to night blindness and peripheral vision loss. Similarly, hearing loss often progresses into the third or fourth decade. By the fourth decade, patients typically approach legal blindness and hearing impairment continues to decline. The proteins that when mutated cause USH are frequently found in primary sensory cells, photoreceptor and hair cells, that directly respond to light and sound, respectively. Similar to other forms of USH, the mRNA coding for the protein responsible for USH type 3 (CLRN1) is expressed in cochlear hair cells of the inner ear. However, as demonstrated in the current study, and unlike other USH disease proteins in the retina, we show that the *Clrn1* is expressed in glial cells in the retina (Müller cells) and is not expressed in the photoreceptors themselves. For reasons that remain unclear, the *Clrn1* knockout mouse does not have a retinal degeneration phenotype but does become deaf soon after birth. In the current paper, we characterize the expression pattern in the retina and analyze the effects of removing the *Clrn1* gene on vision and hearing.

## Introduction

Usher syndrome (USH) is characterized by bilateral combined blindness and deafness, and is the most common cause of the dual sensory deficit [1]. To date, there are 11 chromosomal loci with 9 genes identified, and over 100 mutations directly associated with USH (for reviews see [2]–[4]). Although significant variability is present and misdiagnosis is prevalent [5]–[7], USH has for many years been sub-categorized into USH1, USH2, and USH3, based upon the patient's phenotypic severity and age of onset, as well as the presence or absence of vestibular function [2],[8]. For example, USH1-associated mutations in myosin VIIa, cadherin, protocadherin, harmonin, and SANS result in congenital deafness and progressive retinitis pigmentosa (RP) beginning before puberty. In contrast, mutations in the USH2 genes, *USH2A* and *GPR98*, result in moderate to severe congenital hearing loss, normal vestibular function, and progressive RP. USH3 exhibits progressive post-lingual hearing loss with a late-onset RP [9], which has recently been shown to progress more rapidly than USH2A [10],[11].

Although having the lowest clinical incidence of the three Usher syndromes, USH3 has the greatest therapeutic ‘window’ for treatment due to the relatively late onset and slow progression of vision loss. More importantly in terms of therapy, human USH3 hearing loss is progressive, in contrast to congenital hearing deficits in USH1 and USH2. A single USH3 gene, *CLRN1* (*USH3A*), has been identified [12]–[15] to date, although there is a putative additional locus on chromosome 20 [2],[16]. The incidence of USH3 is increased in Finland [9] and amongst Ashkenazi Jews in various regions [17] due to distinct founder mutations. Twelve *CLRN1* mutations have been documented [6], [10], [12]–[14], [17]–[19], with the two founder mutations being N48K and Y176X [17]; the former amino acid change eliminates the only predicted N-linked glycosylation site, while the latter results in a premature CLRN1 truncation.

Several mouse models of Usher syndrome types I and II have been characterized [20], with only the Usherin null (*Ush2a*) mouse demonstrating a significant retinal degeneration phenotype. The *Ush2a* mutant mouse exhibits a very slow, late-onset retinal degeneration [21]. It remains unclear as to why mutations in USH genes other than Usherin do not result in a retinal degeneration phenotype in mice.

Clarin 1 is a unique protein with homology to other four-pass transmembrane proteins, including members of the Claudin, Connexin, and CACNG protein families [12]. Based on homology between CLRN1 and stargazin, as well as its structural similarity to synaptic cleft and gap junction proteins, CLRN1 was predicted to be localized to the region around specialized ribbon synaptic junctions in both photoreceptor and hair cells. Three recent studies have provided some insight into the primary cochlear defect [22] and CLRN1's cytoskeletal cellular binding partners [23],[24]. In the current study, we characterized the expression pattern of *Clrn1* in the murine retina and analyzed the phenotype of the *Clrn1* KO mouse.

We show here that the loss of CLRN1 results in progressive degeneration and loss of cochlear hair cells and early-onset hearing impairment. Moreover, and in contrast to recent immunohistochemical evidence [24], we find no evidence that *Clrn1* is expressed in murine photoreceptors and no “USH3-like” retinal degeneration phenotype in the *Clrn1* KO mouse. We believe that CLRN1 may represent the first Müller cell-expressed protein which, when mutated, results in an RP phenotype in humans.

## Results

### Molecular Expression of *Clrn1*

**Figure 1:**
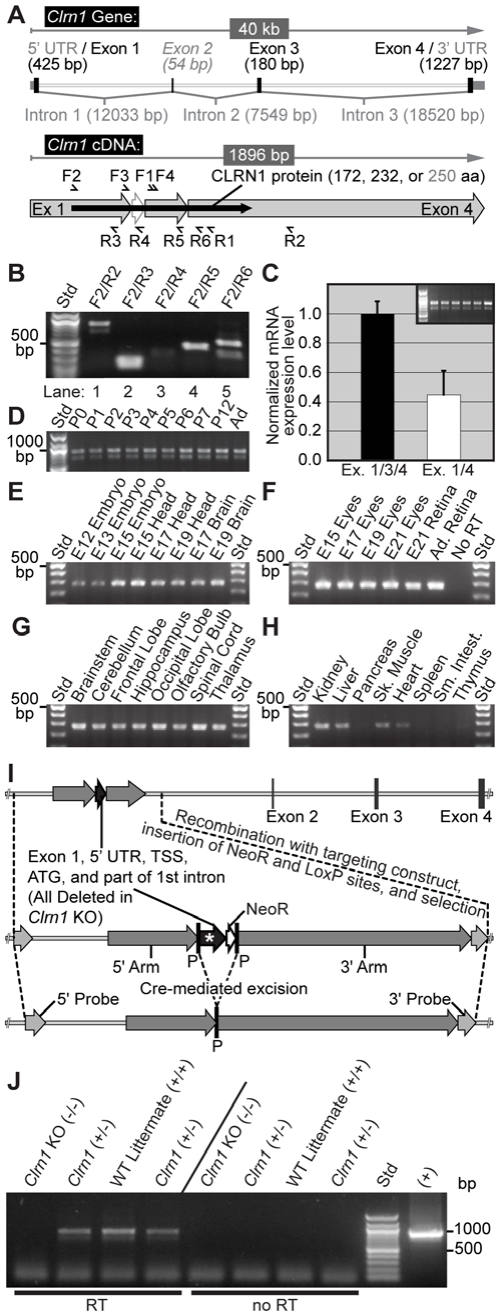
*Clrn1* RT–PCR analysis in mouse retina and KO mouse generation. (A) Schematic diagram illustrating the intron/exon structure of the murine *Clrn1* gene, and the structure of the spliced transcripts showing primer locations. (B) Two transcripts are readily amplified from mouse retina. A common forward primer (F2) was paired with exon-specific reverse primers (R2-R6; see Figure S1) in lanes 1–5. (C) Quantitative RT–PCR analysis comparing major splice variant expression levels in 6 adult C57BL/6J mouse retinas (3 male, 3 female), using F3/R1 primers and fluorescent FAM-BHQ probes (see Figure S1) specific for exon 1/3 or exon 1/4 splice junctions. The inset gel image shows end-point RT-PCR analysis of the same samples using F2/R2 primers. (D) Postnatal developmental and adult expression of *Clrn1* using F2/R2 primers. (E) Prenatal developmental expression of *Clrn1*. Whole embryos, heads, and brain tissue RNAs were amplified using F1/R1 primers. (F) RT–PCR analysis of isolated prenatal eyes, a P21 retina, an adult control retina, and a No RT (no reverse transcription) negative control using F1/R1 primers. (G) Regional expression in the adult mouse brain is demonstrated (F1/R1 primers). All brain regions appear to transcribe the *Clrn1* gene. (H) *Clrn1* expression in adult mouse organs (F1/R1 primers). A more restricted expression profile is evident in mouse tissues outside of the nervous system, similar to expression in human [12]. (I) The 5′ untranslated region (5′ UTR), exon 1 (containing the transcriptional start site (TSS) and the ATG start codon), and the proximal 269 bp of the first intron were removed in embryonic stem cells by homologous recombination. The uppermost construct represents a 50,000 bp genomic fragment on chromosome 3q21, while the two lower constructs represent approximately 20,000 bp centered on exon 1. The genomic region (*) and neomycin resistance gene (NeoR) were spliced out by Cre recombinase activity at loxP (P) sites. (J) RT–PCR analysis of *Clrn1* total KO (−/−), littermate heterozygous controls (+/−), and WT (+/+) animals using F2/R2 primers. No RT = no reverse transcription. (+) = Plasmid DNA containing cloned *Clrn1* cDNA (exons 1, 3, and 4).

RT-PCR analysis of *Clrn1* expression in mouse retina is shown in Figure 1. A diagram of the mouse *Clrn1* gene structure is illustrated in Figure 1A. *Clrn1* transcripts (Ensemble; http://www.ensembl.org/Mus_musculus/Gene/Summary?g=ENSMUSG00000043850) are encoded by four exons spanning 40 kilobases on chromosome 3. Also shown in Figure 1A is the intron-spliced four-exon cDNA structure of the gene. All known transcripts include exons 1 and 4, and the two primary transcripts in the retina and cochlea both lack exon 2 (shown in light gray). The longer transcript includes exons 1, 3, and 4 (NM_153385; http://www.ncbi.nlm.nih.gov/nuccore/68533238), while the shorter splice variant contains exons 1 and 4 only (NM_153386; http://www.ncbi.nlm.nih.gov/nuccore/68533243) (see also [22],[24]). Primers (Figure S1) used to amplify *Clrn1* in all figures are noted above (forward; sense; F1-F4) and below (reverse; antisense; R1-R6) the cDNA diagram. Figure 1B shows adult mouse retinal cDNA amplified with a common *Clrn1* forward (F2) and exon-specific reverse (R2-R6) primers, annealing to each of the 4 exons. Two major *Clrn1* splice variants are readily amplified from retinal cDNA using primers that span all four exons (lanes 1 and 5). The two *Clrn1* splice variants are distinguished by the inclusion of an alternatively spliced exon 3 (180 bp), and previously both transcripts have been shown to be expressed in the mouse inner ear [12],[22],[24]. The “full-length” transcript contains exons 1, 3, and 4 (upper bands, lanes 1 and 5), while the shorter splice variant includes exons 1 and 4 only (lower bands, lanes 1 and 5). Reverse primers annealing to exons 1 (lane 2), 3 (lane 4), and 4 (lane 5) readily amplify *Clrn1* transcripts, whereas a primer annealing to the infrequently included exon 2 (previously referred to as exon 1 [12]) shows very low, but detectable, amplification (lane 3). Together these data indicate that exon 2 is routinely spliced out of the mature transcripts.

Quantitative (q) RT-PCR (Figure 1C, graph) utilizing dual-labeled fluorescent probes that anneal to specific exon-exon junctions (exons 1/3 or 1/4) allowed assessment of the relative expression levels of the two major transcripts. Six retinas from WT C57BL/6J animals (3 males, 3 females) were individually processed for both real-time RT-PCR and end-point (30–40 cycles) RT-PCR. Quantitatively (Figure 1C), the 1/4 exon splice variant (lacking exons 2 and 3) is expressed at approximately 40% of full-length (exons 1, 3, and 4) transcript expression levels. The gel image (Figure 1C, inset) shows an ethidium bromide stained agarose gel reflecting *Clrn1* end-point RT-PCR processed in parallel for the six animals.

*Clrn1* was readily amplified in all postnatal (P) to adult retinal samples throughout mouse development (Figure 1D). Using a pair of primers that span exon 1 (F2) to exon 4 (R2), the two major transcripts were readily detected from the day of birth (P0) through the first postnatal week (P7), as well as in a P12 and the adult mouse. Prenatal *Clrn1* expression was detectable at embryonic (E) day 12 (Figure 1E) using semi-quantitative RT-PCR and primers that span introns. Earlier embryonic stages were not examined. Similar to findings in the inner ear [12], *Clrn1* expression is noted at all developmental ages tested, with slightly higher expression levels observed in the E15 animal (both embryo and whole head samples; Figure 1E). More specifically, embryonic eyes and dissected retinas were examined by RT-PCR (Figure 1F). *Clrn1* is clearly expressed in the eyes (and retinas) of animals before birth. Omission of the reverse transcriptase (RT) enzyme resulted in no amplification (Figure 1F). *Clrn1* expression was also examined in the mouse brain (Figure 1G). All brain regions tested showed *Clrn1* amplification. In contrast to neural and sensory tissue samples, amplification of *Clrn1* transcripts was not apparent in all body tissues or organs (Figure 1H). *Clrn1* was present in kidney, liver, skeletal and heart muscle, and below detection in pancreas, spleen, small intestine, and thymus samples. These data are complementary to previously published data [12]–[14].

### Generation of the *Clrn1* Knockout Mouse

A schematic diagram of the genetic recombination used to generate the *Clrn1* KO mouse is shown in Figure 1I. We eliminated the production of all known coding transcripts in the eye and ear by utilizing Cre recombinase and loxP sites to recombinatorily remove part of the gene promoter, the transcriptional start site (TSS), the entire first coding exon including the 5′ UTR and starting ATG, and a small portion of the 1^st^ intron (collectively labeled with an asterisk; Figure 1I).

Heterozygous animals on a C57BL/6 background were mated and provided viable WT, heterozygous *Clrn1* KO (+/−), and homozygous *Clrn1* KO (−/−) progeny. Figure 1J shows retinal RT-PCR analysis of littermate WT and *Clrn1* KO (homozygous and heterozygous) animals. Using primers (F2/R2) that span from exon 1 to exon 4, both primary transcripts were observed in both WT (+/+) and *Clrn1* heterozygous (+/−) animals, whereas no amplification was observed in the homozygous (−/−) *Clrn1* KO animals. Furthermore, although this was not a quantitative assay, it is apparent that the WT mouse expressed more than either heterozygous animal. Moreover, no amplification was apparent when the RT enzyme was omitted from the cDNA synthesis reaction, ensuring that positive amplification was due to *Clrn1* mRNA expression and not genomic DNA contamination.

### Inner Retinal Expression of *Clrn1*

In contrast to all other known disease-causing mutations resulting in retinitis pigmentosa (RP), *Clrn1* mRNA expression was restricted to the inner nuclear layer (INL) of the retina, and was undetectable in photoreceptor or retinal pigmented epithelial (RPE) cells. This was an unexpected and unusual result, and to the authors' knowledge, this is the first time that a mutant protein produced by the inner retina results in an RP phenotype.

**Figure 2:**
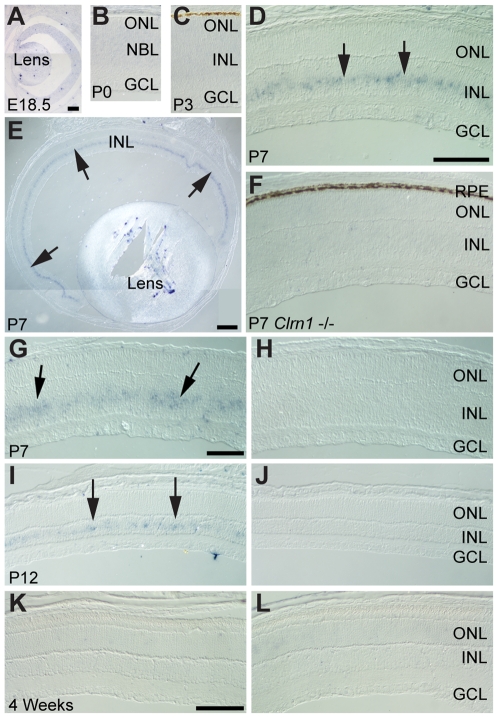
Developmental time course of *Clrn1* in situ hybridization in the mouse retina. (A–C) *Clrn1* was not detected in sections of E18.5 (A), newborn (B), or P3 (C) mouse retina. (D) *Clrn1* in situ labeling in the INL (arrows) of a P7 retina. (E) *Clrn1* in situ showing labeling across the entire central-to-peripheral extent of the retina at P7. (F) Section from a *Clrn1* deficient mouse retina showing lack of labeling. (G,H) *Clrn1* anti-sense (G) and sense control (H) hybridizations showing expression at P7 (arrows). (I,J) *Clrn1* anti-sense (I) and sense control (J) sections from a P12 retina, showing a lower level of expression by this age (arrows). (K,L) *Clrn1* expression at 4 weeks of age. No signal above the sense control (L) could be detected at this age. All sections were hybridized with the same probe and reacted for similar lengths of time. Scale bars = 100 µm for all images except (E), where the scale bar = 200 µm. Scale bars: In A applies to A–C; in D applies to D and F; in G applies to G–J; in K applies to K–L. NBL = neuroblastic layer, CB = ciliary body, ONL = outer nuclear layer, INL = inner nuclear layer, GCL = ganglion cell layer, and RPE = retinal pigmented epithelium.

In situ hybridization was performed with a *Clrn1* riboprobe on developmental retinal tissue from both WT and *Clrn1* KO mice (Figure 2). In WT animals, *Clrn1* mRNA was not detected at E18.5 (Figure 2A), P0 (Figure 2B), or P3 (Figure 2C). However, antisense riboprobe labeling of INL cells was unambiguous at P7 (Figure 2D and 2E). As expected, retinal tissue from the KO animal (Figure 2F) showed no labeling. *Clrn1* ISH was clearly detectable at P7 in the INL (Figure 2G). However, *Clrn1* expression is subsequently down-regulated (Figure 2I) and not detectable by 4 weeks of age in WT mouse retina (Figure 2K), although all ages show expression by end-point PCR (Figure 1). Hybridization with a sense (control) riboprobe showed no labeling at any age (Figure 2H, 2J, and 2L). ISH with a *Clrn1* riboprobe on adult human retina did not generate a significant signal (data not shown). We were unable to obtain human retina at the equivalent stage of fetal development (third trimester) to the peak of labeling observed in the mouse retina (P7).

**Figure 3:**
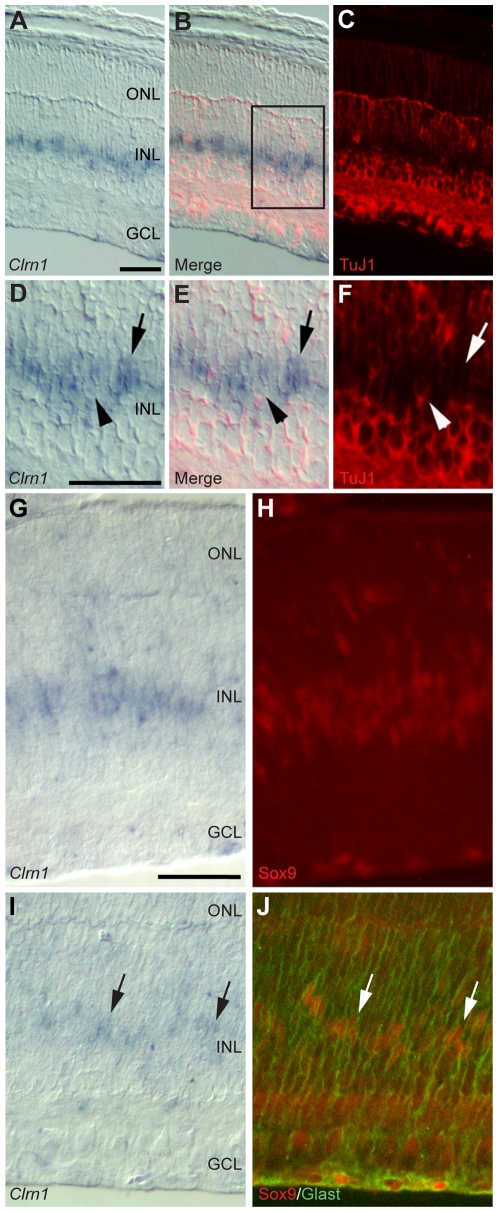
In situ hybridization and immunohistochemistry localize *Clrn1* expression to Müller cells in P7 mouse retina. (A–C) A section of WT retina labeled for *Clrn1* (A) and the neural marker TuJ1 (C). The merged images (B) show the lack of overlap with the neural specific marker. TuJ1 labels all inner retinal neurons, but not the *Clrn1*-positive cells. (D–F) Higher magnification views of the boxed region in (B). Arrowhead points out a TuJ1-positive cell that is not *Clrn1*-positive, while the arrow points to a cell that is *Clrn1*-positive but not TuJ1-positive. (G,H) Peripheral retina labeled with the *Clrn1* riboprobe (G) and subsequently with an antibody to Sox9 (H), a marker of retinal progenitors and Müller glia. We find double labeled cells throughout the peripheral retina. (I) In the central retina, where the last progenitors are turning into Müller glia, a thinner row of *Clrn1*-positive cells remains. (J) The section shown in (I) was double immunolabeled (after ISH) with antibodies to Glast (green) and to Sox9 (red). Arrows (I and J) identify triple-labeled Müller cells. Scale bars = 50 µm in all images. Scale bars: In A applies to A–C; in D applies to D–F; in G applies to G–J. ONL = outer nuclear layer, INL = inner nuclear layer, GCL = ganglion cell layer.

To follow up on inner retinal localization of *Clrn1* by ISH in WT retinal tissue (Figure 3), we performed immunohistochemistry to clarify which retinal cell type(s) express *Clrn1*. The blue-purple ISH reaction product represents *Clrn1* mRNA, whereas red and green fluorescence indicates immunohistochemical staining. An antibody (TuJ1) to tubulin type III labeled all neuronal elements in the retina (shown in red; Figure 3B, 3C, 3E, and 3F). Dual labeling indicates that TuJ1 and *Clrn1* lack co-localization, and thus are not co-expressed in neuronal cells. We also double-labeled P7 retinal sections for *Clrn1* ISH and Sox9 immunohistochemistry (Figure 3G–3J). Sox9 is a transcription factor that is widely expressed in retinal progenitor cells during development. Sox9 expression is maintained in Müller cells but lost in maturing neurons [25]. In peripheral retina, the antibody to Sox9 (red) labeled a band of retinal progenitor and Müller glia (Figure 3G and 3H). In the more differentiated central retina, a narrower band of *Clrn1* ISH-positive cells (Figure 3I) is apparent, and some of these cells also stain positively for Sox9 (Figure 3J; red) and Glast (Figure 3J; green). These data add additional support to the identification of Müller cells as the predominant source of *Clrn1* expression in the retina. Additionally, staining of P7 mouse retina shows co-labeling of *Clrn1* ISH with a Glast antibody (Figure S2). Double labeling with Glast, a Müller glial cell marker, indicates that some Glast-positive cells also express *Clrn1* mRNA. Surprisingly, these data show that *Clrn1* mRNA (and presumably the protein product(s) of the *Clrn1* gene) is expressed in retinal glia and not in neurons, including photoreceptors.

**Figure 4:**
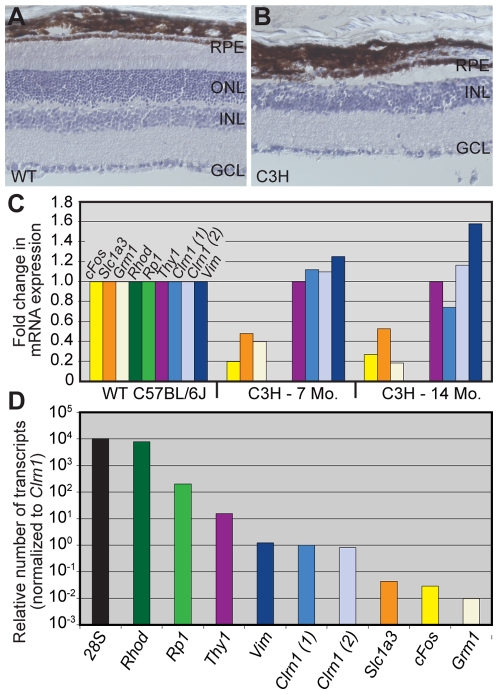
Analysis of *Clrn1* in WT and photoreceptorless C3H/HeJ aged mouse retinas. (A,B) Sixteen micron cryosections of WT (A) and degenerated C3H (B) mouse retinas counterstained with hematoxylin. Note the virtual complete loss of photoreceptors in the 14 month C3H retina. (C) Real-time quantitative (q) RT–PCR was performed in order to correct for the RNA contribution of the outer retina in the WT cDNA sample. The data were normalized to the ganglion cell marker *Thy1*, and subsequently to expression levels in the WT sample. For comparison purposes, several additional known retinal transcripts were included in the qRT–PCR analysis. (D) Relative transcript expression levels in retinas from WT and photoreceptorless animals. Normalizing to a *Clrn1* primer pair, the range in the abundance of non-Clrn1 transcripts was 100-fold fewer (Grm1) to 10,000-fold higher (*Rhod*). Scale bar = 50 µm in all images. ONL = outer nuclear layer, INL = inner nuclear layer, and GCL = ganglion cell layer.

**Figure 5:**
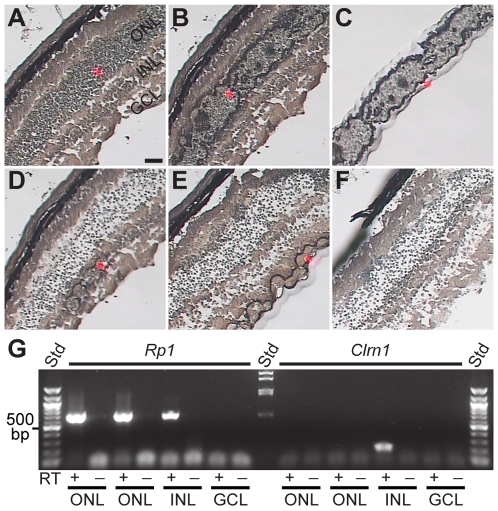
Laser capture microdissection of WT mouse retina. (A–F) Images of a section of WT C57BL/6J retina taken during the laser capture process. (A) Retina before laser capture. (B) The ONL is being captured on the cap. (C) The ONL after capture, on the surface of the microfuge tube cap just prior to total RNA extraction. (D) The ONL has been removed, and the INL is being laser-captured. (E) The ONL and INL have both been removed (though some cells remain), and the GCL (ganglion cell layer) is being laser captured. (F) Retina remaining after cell layers have been laser captured and collected on microfuge tube caps. (G) RT–PCR was used to amplify *Rp1* (a photoreceptor specific transcript) and *Clrn1* in each sample. *Rp1* was detected in both ONL samples as well as the INL sample due to carry-over of a few photoreceptors, but was not detected in the GCL. Note that *Clrn1* transcripts were only detected in the INL. Scale bar = 50 µm. ONL = outer nuclear layer, INL = inner nuclear layer, GCL = ganglion cell layer, and RPE = retinal pigmented epithelium.

We performed two additional experiments (Figure 4 and Figure 5) in order to validate our ISH data shown in Figure 2 and Figure 3. We first used a genetic approach and examined a well-studied model of photoreceptor degeneration: the C3H/HeJ (C3H) mouse, which harbors the photoreceptor *Pde6b^rd1^* mutation [26]. The mutation results in a rapid degeneration and loss of nearly all photoreceptor cells [27],[28]. A histological comparison of a 6-month-old WT C57BL/6J and a 14-month-old C3H mouse is shown in Figure 4A and 4B, respectively. Note the maintenance of the GCL and INL, but near complete loss of the ONL in the C3H animal (Figure 4B). We performed qRT-PCR and normalized the WT and C3H samples to the ganglion cell-specific transcript, *Thy1*, as well as to *28S* RNA. We also measured the expression levels (Figure 4C) of six genes in addition to *Clrn1* and *Thy1*: *cFos, Slc1a3, Grm1, Rho, Rp1*, and *Vim*. When compared to expression levels in the WT animal, the loss of photoreceptors resulted in reduced expression of the immediate early gene transcription factor *cFos*, as well as *Slc1a3* and *Grm1*, a glutamate transporter and receptor, respectively. Not surprisingly, extremely low levels of the photoreceptor-specific transcripts *Rho* and *Rp1* were measured in the C3H mutants. These data confirm our histological observations that photoreceptors are for all intents and purposes absent or completely degenerated in the C3H animals. Importantly, as measured with two independent primer pairs, *Clrn1* exhibited essentially no difference in expression in the C3H retinas (Figure 4C; *Clrn1* (1), *Clrn1* (2)) compared to the WT sample. The vimentin (*Vim*) transcript (Figure 4C) appeared to be slightly up-regulated in both degenerated C3H retinas, a finding consistent with other models of photoreceptor degeneration [29]. Figure 4D shows relative WT expression levels of the *28S*/*Thy1*-normalized transcripts (Figure 4C). In Figure 4D, the averaged raw Ct (threshold cycle number) data for each transcript were normalized such that *Clrn1* expression was assigned an expression level of 1. Though not quantitative, these data provide approximations of the relative abundance of genes in WT animals compared in Figure 4C. Hence, *28S* and *Rho* express roughly 10,000 and 8,000 times the number of *Clrn1* transcripts, respectively. *Rp1*, another photoreceptor-specific transcript expresses about 200-fold higher levels, while *Thy1* expresses about 15 times the number of *Clrn1* transcripts. Finally, *Vim* mRNA is expressed at a roughly similar level, while significantly fewer *cFos*, *Slc1a3*, and *Grm1* transcripts are expressed in the WT mouse retina.

A similar genetic experiment (Figure S3) was performed using the Proline-23-Histidine line 3 (P23H-3) rhodopsin mutant and Royal College of Surgeons (RCS) mutant rats [30],[31]. Analogous to the mouse, histological analysis shows a near complete loss of photoreceptors in the P23H-3 rat (Figure S3B) compared with the WT retina (Figure S3A). In both rat retinal degeneration models *Rp1* expression is almost completely lost, indicating a near total loss of photoreceptors (Figure S3C and S3D). When equivalent total amounts of whole retinal cDNA were PCR amplified (without normalization), P23H-3 and RCS retinal samples show a relative increase in *Clrn1* expression compared to control retinas. The apparent increase in *Clrn1* expression is due to the loss of the photoreceptor RNA contribution to the total RNA samples from mutant animals when equal amounts (from WT and mutant retinas) were included in the amplification reactions. This apparent change in expression is quantified by qRT-PCR (Figure S3E) and represents the average of two retinas. These data suggest that approximately 75% of total retinal RNA in WT animals is produced by photoreceptors, resulting in a ∼4-fold apparent increase in *Clrn1* expression when photoreceptors are absent from the mutant retinal samples.

In a complementary experiment, we used LCM (Figure 5) to isolate mRNAs from individual nuclear layers from WT mouse retinal sections. Micrographs (Figure 5A–5F) show the progression of laser capture sample collection. Red spots (Figure 5A–5E) show the location of the laser beam used to select each of the three individual nuclear layers: ONL, INL, and GCL. Figure 5A shows the retinal section before laser irradiation and Figure 5B shows the retina after the tissue was laser-selected (but not removed). Following removal, the selected/collected tissue (photoreceptors; ONL) was observed on the underside of the tube cap (Figure 5C). Figure 5D and 5E represent capture of the INL and GCL, respectively, while Figure 5F indicates the retinal tissue that remained after the collected cells were physically removed from the tissue section. Using LCM, we collected total RNA from each of the three major retinal nuclear layers (ONL, INL, GCL), and subsequently subjected the isolated total RNA to RT-PCR analysis (Figure 5G). Total RNA was extracted and amplified using two rounds of RT-PCR for *Rp1* (a photoreceptor specific transcript) and *Clrn1* (Figure 5G). As expected, robust *Rp1* expression was detected in both ONL samples and absent in the GCL. We believe that the presence of some *Rp1* transcripts in the INL sample is the result of a few photoreceptors inadvertently adhering to the cap of INL sample. Nevertheless, *Clrn1* was only observed in the INL sample, and was completely absent from both ONL and GCL samples, further supporting the restricted INL localization of *Clrn1* transcripts (Figure 2, Figure 3, and Figure 4).

### Retinal Structure in the *Clrn1* Knockout Mouse

**Figure 6:**
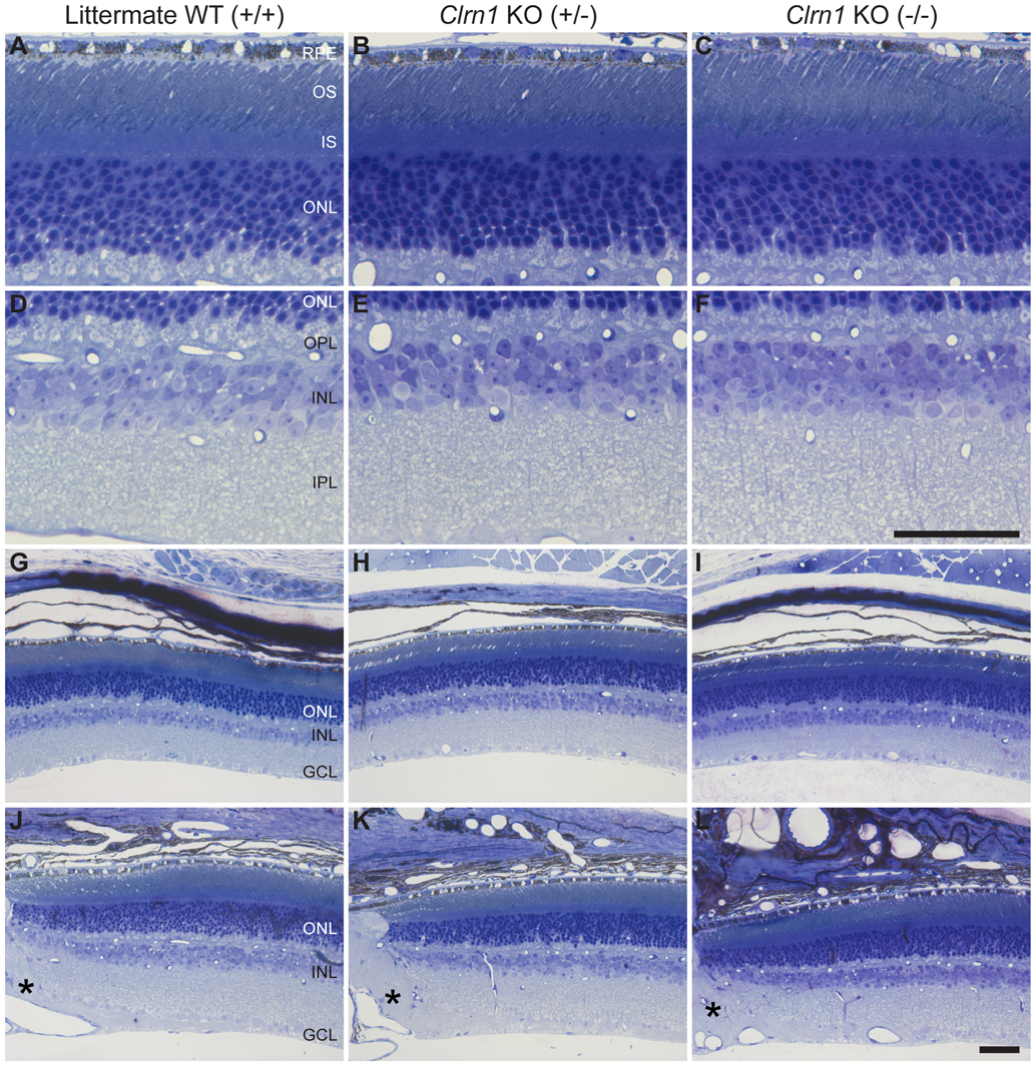
Histological analysis of *Clrn1* KO retinas at 9 months of age. (A–C) Compared to WT animals (A), outer retinal histology appears normal in heterozygous (B) and homozygous (C) *Clrn1* KO mice. (D–F) Similarly, the inner retina of the *Clrn1* KO (+/−,−/−) animals is indistinguishable from the WT animal (D). (G–L) When compared to WT (+/+) animals (G and J) at lower magnification, gross retinal structure seems unperturbed by the lack of *Clrn1* expression (H,I,K,L) in either mid-peripheral retina (G–I) or central retina (J–L) immediately adjacent to the optic nerve head (asterisks). Scale bar = 50 µm. ONL = outer nuclear layer, IS = photoreceptor inner segments, OS = photoreceptor outer segments, and RPE = retinal pigmented epithelium.

*Clrn1* KO mice show no phenotypic signs of retinal degenerative disease. Since the USH3 human combined blindness and deafness phenotype is progressive, we studied *Clrn1* KO mice up to 2 years. At 2 years of age, *Clrn1* KO mice continued to exhibit a normal retinal phenotype, both structurally and functionally. Figure 6 is toluidine blue stained 1 µm plastic embedded sections of 9 month old WT (+/+) and *Clrn1* mutant (+/−,−/−) animals. Figure 6A–6C show high magnification of the photoreceptors and their associated RPE, while Figure 6D–6F show the inner retina at the same magnification. Lower magnification offers a more gross histological view of mid-peripheral (Figure 6G–6I) retina, as well as retina immediately adjacent to the optic nerve head (asterisks, Figure 6J–6L). No abnormal histological phenomena were detected.

**Figure 7:**
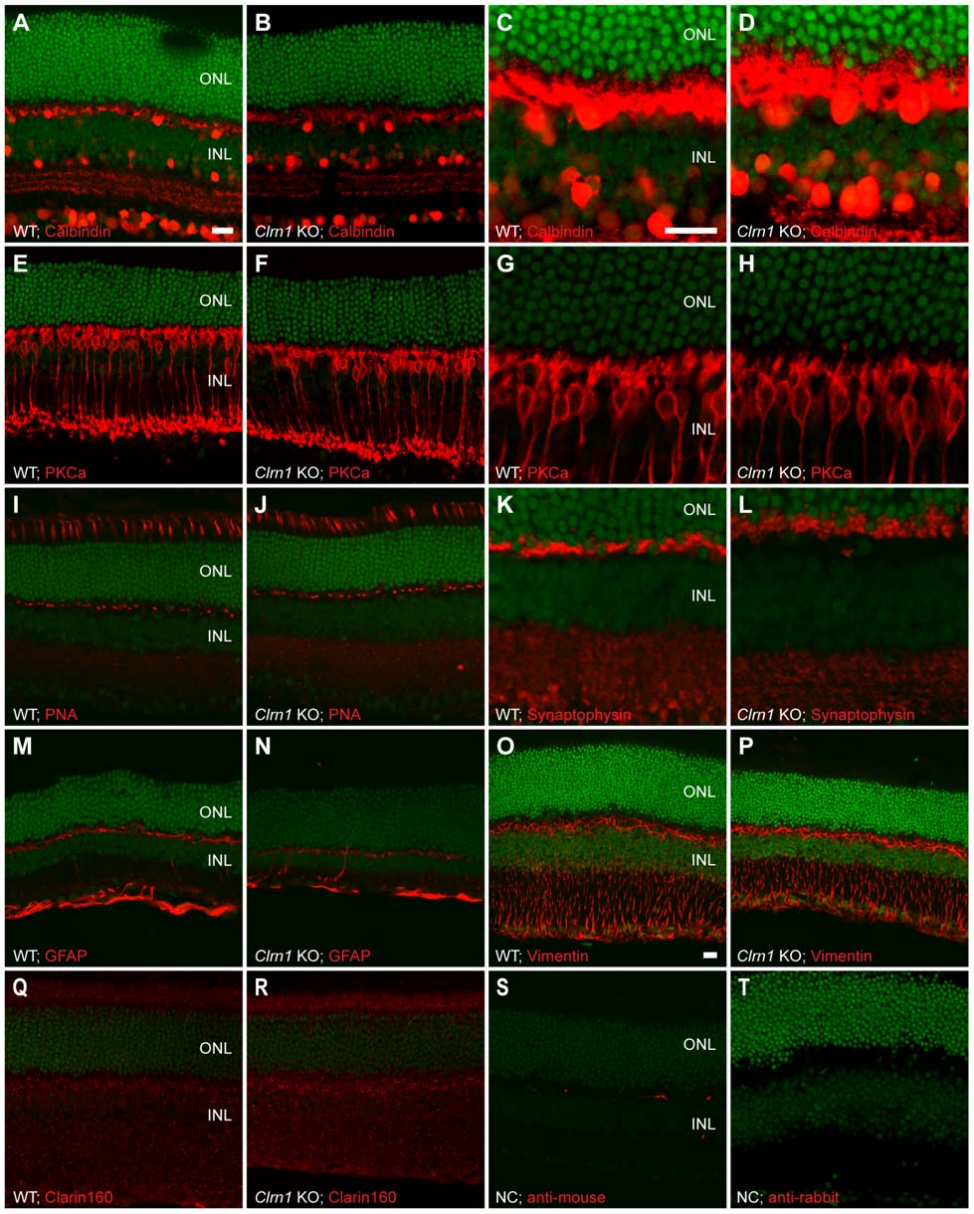
Immunohistochemical analysis of littermate WT (+/+) and *Clrn1* KO (−/−) mouse retinas. (A–D) WT and KO retinas stained with an antibody to Calbindin, shown at low (A and B) and high (C and D) magnifications. (E–H) WT and KO retinas stained with an anti-PKC-alpha antibody at low (E–F) and high (G–H) magnifications. (I,J) WT and KO retinas labeled with the rhodamine-conjugated peanut agglutinin (PNA) lectin. (K,L) WT and KO retinal sections immunostained with anti-Synaptophysin antibodies, shown at high magnification. (M–P) WT and KO retinal sections stained with intermediate filament antibodies to GFAP (M,N) and vimentin (O,P). (Q,R) Immunostaining with a candidate antibody made to CLRN1 (the *Clrn1* gene product) in WT (Q) and KO (R) retinas. (S,T) Control images showing labeling by the secondary antibodies alone. Scale bar = 20 µm in all images. Scale bars: In A applies to A–B, E–F, I–J, M–N, and Q–T; in C applies to C–D, G–H, and K–L; in O, applies to O–P. INL = inner nuclear layer, ONL = outer nuclear layer. SYTOX Green was used as the nuclear counterstain.

We performed fluorescent confocal microscopy using a panel of antibodies to look for subtle structural or cellular changes in the *Clrn1* KO retina (Figure 7). Eighteen month old littermate animals (WT and KO (−/−)) from early heterozygous matings were used. Antibodies used were: anti-Calbindin (horizontal and amacrine cells; Figure 7A–7D), anti-PKCa (rod bipolar cells; Figure 7E–7H), Rhodamine-PNA (cone photoreceptor sheaths; Figure 7I and 7J), anti-synaptophysin (OPL and IPL synaptic structures; Figure 7K and 7L), anti-GFAP and anti-vimentin (glial cells; Figure 7M–7P), and a chicken anti-CLRN1 antibody (Clarin160; Figure 7Q and 7R. See below, and Figure S4 and Figure S5). No differences between WT and *Clrn1* KO (−/−) animals were found using these antibodies. Due to low levels of retinal CLRN1 expression, the anti-CLRN1 antibody labeling (Figure 7Q and 7R) represents non-specific signal in both WT and *Clrn1* KO retinas. Labeling with the secondary antibodies alone showed no reactivity (Figure 7S and 7T). It is important to note that when an EGFP-CLRN1 fusion protein is overexpressed in 293 cells (see below, and Figure S4), specific labeling is readily observed using two different anti-CLRN1 antibodies, including the Clarin160 antibody immunoreactivity shown here (Figure 7Q and 7R).

### Clarin 1 Antibody Analysis and Membrane Localization in Transfected Cells

Using the pEGFP-C1 vector (Clontech), we generated and expressed the EGFP-CLRN1 fusion protein in HEK293 cells (Figure S4), driven by the human cytomegalovirus (CMV) immediate early promoter. Transfection with the vector alone resulted in EGFP expression localized throughout cell somata (Figure S4A). CLRN1 (containing exons 1, 3, and 4) was fused to the 3′ end of EGFP, and following expression the resultant fusion-protein was redistributed and concentrated to plasma membranes (Figure S4B). This finding supports previous predictions based upon the primary structure[12], as well and recent studies [23], that full-length murine CLRN1 (232 aa) is predominantly a plasma membrane-targeted protein.

Anti-CLRN1 antibodies were screened for specificity using EGFP-CLRN1 transfections in HEK293 cells. With the red channel turned off, clear EGFP-CLRN1 fluorescence is observed in a subset of cells (Figure S4C). Red fluorescence (Figure S4D) shows subsequent immunostaining with an anti-CLRN1 affinity-purified antibody (CLRN1-B2). The CLRN1-B2 antibody (rabbit) showed precise co-localization (Figure S4E) with the EGFP-CLRN1 fusion protein. In a similar experiment the Clarin160 antibody (chicken), which was generated against a different CLRN1 peptide, showed equally reliable co-localization with EGFP-CLRN1 (Figure S4F, S4G, S4H). No immunoreactivity (red) was detected when the primary antibody was omitted (Figure S4I, S4J, S4K) or in non-transfected cells (data not shown). Several additional anti-CLRN1 antibodies showed various degrees of specific/non-specific labeling (data not shown).

Western blot analysis (Figure S4L) of adult human, mouse, and rat retinal homogenates using the CLRN1-B2 antibody detected a band at ∼25–26 kDa in all species, and an additional ∼35 kDa protein in the human sample only. Detection was dependent upon sodium dodecylsulfate (SDS) inclusion in the sample buffer, as no reactivity was observed in samples processed without SDS (Figure S4L, right side). We immunoprecipitated CLRN1 from mouse retinas using the CLRN1-B2 antibody to further characterize the CLRN1 protein (Figure S4M). After elution and subsequent immunoblotting with the same antibody, a higher molecular weight (∼55–80 kDa) protein complex was detected prominently in the first two eluates (lanes 1 and 2). These data indicate that CLRN1 can homodimerize and/or participate in the formation of larger multi-protein complexes under non-reducing conditions. Studies to identify CLRN1 interacting proteins provide additional insight into CLRN1 function [23], and there have been indications that CLRN1 associates with both actin [23] and microtubules [24].

Immunocytochemistry on P7 WT mouse retinal sections (Figure S5), the peak of *Clrn1* mRNA expression as detected by ISH (Figure 2), failed to show specific CLRN1 localization using Clarin160 (Figure S5E, S5F) or CLRN1-B2 (Figure S5G, S5H) antibodies. Similar labeling was observed in both WT and *Clrn1* KO control retinal sections for each of the antibodies tested.

### Retinal and Cochlear Function

Recent data suggest that *Clrn1* KO mice are functionally normal [22]. We independently performed ERG experiments (Figure S6) in order to measure photoreceptors A- and B-wave function in WT and *Clrn1* KO animals up to 2 years of age. Photoreceptor function in the *Clrn1* KO animal appeared normal, and no significant difference was observed at any light intensity. In contrast to the lack of a retinal phenotype, but similar to most other mouse models of Usher syndrome [20], the *Clrn1* KO mouse exhibits significant structural and functional hearing deficits [22]. Similarly, we demonstrate inner ear dysfunction in the *Clrn1* KO mouse by measuring the auditory brainstem response (ABR). Figure S7 illustrates representative ABR traces, and corroborates the functional loss in both juvenile and adult animals [22].

### Outer and Inner Hair Cells Degenerate in *Clrn1* KO Mice and Exhibit Longer Stereocilia

**Figure 8:**
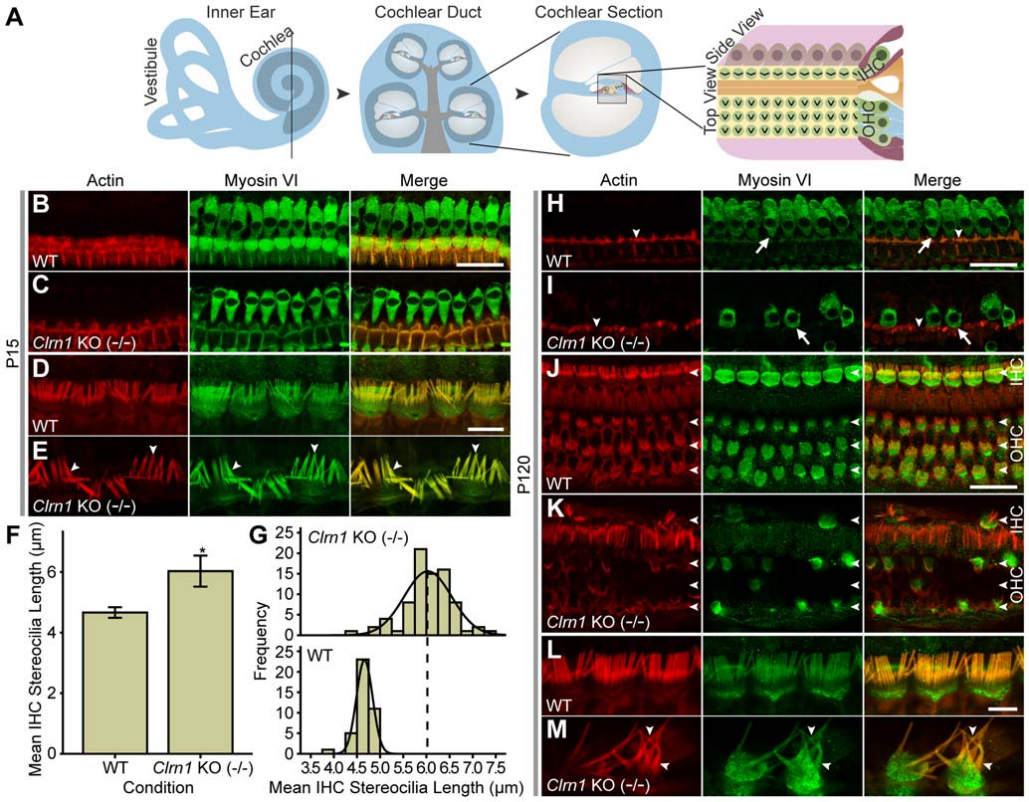
Cochlear hair cells of *Clrn1* KO (−/−) mice degenerate with age and exhibit longer stereocilia. (A) Illustration of the inner ear, demonstrating the cochlea and auditory sensory epithelium. (B–E) Immunofluorescent confocal images of P15 and mouse inner-ear epithelia harvested from the middle turn of the cochlea, shown in side view. Filamentous actin was detected with rhodamine-Phalloidin (red), and anti-myosin VI labeling is shown in green. (B,C) At P15, the auditory epithelium in *Clrn1* KO (−/−) (C) mice appears morphologically similar to WT animals (B). (D) WT animals maintain organized stereocilia on inner hair cells (IHCs). (E) Subtle IHC stereociliar abnormalities such as increased bundling and lengthening (arrowheads) are evident in KO animals at P15. (F,G) Quantitative analysis of IHC bundle lengths reveal considerably longer stereocilia (F) in P15 *Clrn1* KO animals, distributed over a broader range of lengths (G, upper graph; n = 80), which is distinguishable from the highly uniform lengths of WT stereocilia (G, lower graph; n = 40). (H,I) Shown from the side view at P120, and compared to WT labeling (H), staining of *Clrn1* KO auditory epithelia (I) indicates extensive degeneration and loss of IHCs. At this age, a disruption of supporting cell apical surfaces is apparent (I, arrowheads), and the surviving IHCs in the auditory epithelia of *Clrn1* KO mice exhibit shortened hair cell bodies (I, arrows). (J,K) From a top view, a clear disruption and loss of both IHCs and OHCs is evident in the *Clrn1* KO mutant (K, arrowheads). (L,M) IHCs from WT cochleas (L) possess highly ordered and symmetrical stereocilia, while *Clrn1* KO IHCs (M) exhibit profoundly disorganized bundles of elongated and possibly fused stereocilia (arrowheads). * = P<0.001. Error bars = 1 SD. Scale bars: In B (25 µm) applies to B and C; in D (7.5 µm) applies to D and E, in H (25 µm) applies to H and I, in J (20 µm) applies to J and K, in L (5 µm) applies to L and M.

Immunofluorescence with a hair cell-specific marker, myosin VI, and filamentous actin were used to evaluate the cellular arrangement and general morphology of the auditory sensory epithelium of WT and *Clrn1* KO (−/−) mice (Figure 8). Recently Clrn1 was shown to be directly associated with the cellular actin networks [23], so staining for f-actin is particularly interesting in the *Clrn1* KO animals. Figure 8A illustrates the origin of the cochlear tissue depicting the planes of section used in the analysis of the organ of Corti (middle and apical turns; side and top views). At P15, there is no apparent cell loss in the organ of Corti (Figure 8B and 8C), however, the mechanosensory stereocilia, the actin-rich projections on the apical surface of the inner hair cells (IHC) appear disorganized, elongated, and variable in length (6.03±0.51 µm, n = 80, P<0.001) when compared to the WT control (4.66±0.174 µm, n = 80) (Figure 8D–8G). Similar non-quantitative morphological changes in stereocilia have been noted previously [22]. At P120, the cochlear outer hair cells (OHC) and IHC, as well as the supporting cells, are severely degenerated (Figure 8H–8M). By P120, the number of cell bodies is significantly reduced, and the remaining cells have reduced apical surfaces and display bundles of severely disorganized and elongated stereocilia (Figure 8M). The supporting pillar cells, two closely adjacent rows of epithelial cells that separate the inner and outer rows of hair cells, appear structurally compromised, as indicated by a reduction in rhodamine-Phalloidin staining of the pillar heads (Figure 8I).

Some *Clrn1* KO mice on a C57BL/6 background exhibited circling behavior by 5–6 months of age (Video S1). Circling behavior was more prevalent when the KO construct was backcrossed to a mixed C57BL/6 and Sv129 genetic background (data not shown). However, the circling phenotype remained incompletely penetrant, irrespective of the genetic background, consistent with the variable finding of vestibular defects among USH3 patients. We analyzed filamentous actin features of utricular cells in vestibule of the randomly selected (non-circling) 4-month WT and *Clrn1* KO animals on the C57BL/6 background (Figure S8). Neither low (Figure S8A, S8B) nor high (Figure S8C, S8D) power images indicate any grossly abnormal structural features in the vestibule at P120.

## Discussion

Photoreceptors are known to express over 300 unique (within the retina) transcripts [32], many of which are unlikely to be expressed outside of the eye. This finding is consistent with clinical disease manifestations, where mutations (dominant or recessive) in rod photoreceptor cell-expressed genes commonly result in photoreceptor cell death and consequent RP. There are documented examples of RPE-defects [33],[34], as well as a predominantly expressed ganglion cell transcript [35] that result in an RP-like phenotype. To date, retinal degeneration resulting from mutation in proteins originating from neurons or glia of the inner nuclear layer has not been described. Our data suggest that CLRN1, the protein implicated in Usher syndrome type 3 which caused progressive blindness and deafness in humans, is an inner retinal protein (at least in mice) produced by the retinas resident radial glia, Müller cells.

Retinitis pigmentosa (RP) is a group of blinding diseases affecting approximately 1.5 million people worldwide, and around 1 in 4000 births in the United States [36]–[38]. RP is sometimes referred to as rod-cone dystrophy, and is characterized by a loss of rod photoreceptor cells, narrowing of the visual field, and night blindness, which can eventually progress to cone dysfunction and total vision loss. Thus far, over 40 loci have been identified for non-syndromic RP [38] and 30 genes have been causally related (RetNet; http://www.sph.uth.tmc.edu/Retnet/). In contrast, cone or cone-rod dystrophies (21 loci, 15 identified genes) are due to primary defects in cone cells, and in turn, rods may eventually become vulnerable to progressive degeneration [39]. Usher syndrome (USH) is fundamentally characterized by combined deaf-blindness. There are over 50 syndromic/systemic loci involving RP (RetNet), about 25% of which are also associated with USH. USH syndromes are the most common cause of this combined deaf-blindness, accounting for approximately 50% [1],[16], while contributing to about 14% of all RP cases [34]. Nine of the 11 Usher syndrome loci have been genetically characterized, with 2 genes (USH1E and USH1H) not yet identified [4].

Numerous transgenic and knockout mouse models of RP have been developed, in which a mutation or knockout of a photoreceptor or RPE specific gene results is an approximation of the human clinical phenotype, often exhibiting an abbreviated time course; among these are Rho [40], Pde6b [41], RDS [42], Rom1 [43], Crx [44], Tub [45], Gucy2e [46], RPE65 [47], and others. To date, knockout of Usher gene product has been largely unsuccessful in generating animal models with a retinal degeneration phenotype in mice, with only the Usherin null (*Ush2a*) mouse demonstrating a significant disease. [20],[21]. Surprisingly, the majority of these USH animal models exhibit pronounced hearing defects [48]–[52]. Small alterations in the ERG amplitude and waveform have been noted in some of these mice [53]–[55] but the implications of these changes remain unclear. Overall, relatively few tetraspanins have been identified as having essential functions, and mouse knockouts of individual tetraspanins has resulted in mild phenotypes [56].

The lack of genotype-phenotype correlation in the retina of USH mouse models remains unclear. In the absence of direct evidence of a redundant pathway in mice, it is reasonable to suggest that there is compensation via undiscovered gene products and/or intracellular pathways mitigating the loss of USH gene expression in the mouse retina. USH proteins in the retina are generally thought to be localized to photoreceptor and/or RPE cells. USH proteins are commonly associated with photoreceptor connecting cilia, inner/outer segments, and synaptic regions [2],[3],[16]. Indeed, recent immunohistochemical evidence purports CLRN1 to be localized to photoreceptors [24], and it remains unclear how to harmonize these data with our findings presented here, where we show that *Clrn1* is not expressed in photoreceptors. Nevertheless, it has been proposed that USH proteins form a network of directly associated proteins within the cell, localizing at or near the photoreceptor's connecting cilium, at the apical part of the inner segment [3],[16],[57],[58]. It follows then that damage to any one of the proteins within the complex could theoretically result in clinically similar USH-related RP phenotypes. Though some USH gene products localize to the synaptic terminal, there is little evidence for synaptic zone dysfunction in human electrophysiological recordings [10],[59]. Indeed, our original hypothesis maintained that CLRN1 would be a protein facilitating structural maintenance of the synaptic connection between photoreceptors and second order neurons, horizontal and bipolar cells [12]. However, this hypothesis is not supported by data presented here.

### Developmental Expression of the Murine USH3A Gene *Clrn1*

We demonstrated by RT-PCR that *Clrn1* is readily amplified from a variety of adult mouse tissues, all examined brain regions, and the retina. Two primary transcripts were expressed at an approximate 5∶2 ratio; the primary transcript includes exons 1, 3, and 4, while lower levels of expression are observed for the splice variant including exons 1 and 4. This is consistent with the expression of *Clrn1* splice variants in the inner ear [12]. The purpose of inclusion/exclusion of exons 3 and/or 2 in mature *Clrn1* transcripts remains unclear; as is the significance of *Clrn1* splice variant expression levels. Nevertheless, the expression levels of the splice variants appear to be consistently regulated. Based on topological predictions [12] the shorter splice variant (without exon 3) would lack two of the four predicted transmembrane domains if translated into a functional protein. Thus, it is interesting to consider the possibility that the shorter splice variant may exhibit distinct biophysical properties and/or functions from its longer, four transmembrane “full-length” counterpart.

Our results show that although *Clrn1* mRNA can be readily RT-PCR amplified from embryonic, juvenile and adult retina, the mRNA is developmentally down regulated in adult mice. These data suggest that CLRN1 (protein) expression is likely to decrease over the course of retinal maturation, and that CLRN1 is probably translated and expressed at comparably low levels in the adult mouse. It may be that CLRN1 is non-essential to the adult mouse retina, and that CLRN1 serves only a transient (and presumably non-deleterious) role during murine retinal development. We do not currently have *CLRN1* expression data from developing or adult human retina. However, it is intriguing to postulate that the *CLRN1* gene may be differentially regulated in humans. If, in fact, human regulation differs from the mouse with respect to *Clrn1* gene expression, and human retina exhibits (or requires) robust and/or continuous expression into adulthood, it seems reasonable to postulate that humans may develop the characteristic age-related USH3 retinal phenotype whereas mice do not. In other words, humans, but not mice, may have maintained and/or evolved a need for continuous CLRN1 expression in the retina. Another important possibility to consider is that mice, but not humans, express a compensatory protein or utilize a redundant functional pathway that obviates the need for CLRN1 in the mouse retina. Finally, it also seems reasonable to suggest that degeneration in the mouse retina could be dependent upon the expression of gain-of-function mutant CLRN1 proteins, and not simply the loss of CLRN1 altogether, as in the case of the total KO. In this case, the mouse cochlea would remain sensitive to either the absence (KO) or mutation (point mutation or truncation) of CLRN1, whereas the retina would be insensitive in the KO condition, but would degenerate when mutant CLRN1 proteins are expressed. Further studies of animal models exhibiting specific gene alterations, as well as analysis of gene expression in humans, should help to clarify the normal and pathological roles of CLRN1 in these sensory tissues.

### The USH3A Gene, *Clrn1*, Is Expressed in Müller Cells in the Inner Retina

In situ hybridization and laser capture microdissection indicate that *Clrn1* is expressed in the INL, and not in the ONL (photoreceptors), as our original hypothesis proposed. Further supporting this unusual finding is the fact that aged animals (C3H mice, RCS rats, and P23H-3 rats) that have completely lost their photoreceptors continue to express *Clrn1* at WT levels. If these data hold true in humans, they imply that CLRN1 is expressed in the INL and that proteins expressed in the inner retina, and specifically in Müller cells, can cause RP.

The expression of *Clrn1* in Müller cells was surprising. All three sub-classifications of USH (types I, II, and III) exhibit late-onset retinitis pigmentosa (RP). This phenotype, in general, suggests that a slow but perpetual rod photoreceptor degeneration underlies disease progression. Our original hypothesis stated that *Clrn1* would be expressed in photoreceptors, and that the protein, CLRN1, would be targeted to rod photoreceptor synapses and contribute to synaptic adhesion and/or structure. Although significant variability exists among affected individuals, on average, USH3 patients exhibit a 50% functional vision loss at a mean age of 28 [11] and commonly have “tubular vision” by the age of 30 [9]. This is in contrast to numerous other recessive RP (or rod-cone) diseases, in which the onset is often in the first to second decade of life [38]. The discovery that *Clrn1* is expressed in a developmentally regulated manner in Müller cells, and that *Clrn1* is not expressed in photoreceptors, was unexpected. Taken together, our data suggest that an INL protein (CLRN1), when mutated in humans (but not mice), results in degeneration of rod photoreceptors. Assuming that *Clrn1* is similarly localized to Müller cells in the human retina, we believe this to be the first instance of an INL transcript resulting in inherited photoreceptor degeneration [10].

### CLRN1 Protein Expression

Immunocytochemical detection of CLRN1 protein in retinal tissues has not been successful. Two antibodies (one developed in rabbit, one in chicken) accurately detected an EGFP-CLRN1 fusion protein transiently expressed by HEK293 cells (Figure S4). Moreover, the CLRN1-B2 antibody specifically detected protein bands at approximately the correct (predicted) molecular weight(s) in human, rat, and mouse retinal homogenates (Figure S4L). Immunoprecipitation (IP) with the CLRN1-B2 antibody indicates that CLRN1 may form higher molecular weight multimers and/or participate in a multi-protein complex, similar to complexes previously proposed [16],[60], although none of the other USH-related proteins appear to be co-expressed in Müller glia.

In side-by-side comparisons with *Clrn1* KO tissue samples, neither of our CLRN1 antibodies that demonstrated good specificity *in vitro* (Figure S4) offered a reliable immunohistochemical signal in WT retinal tissue sections (Figure S5). Similar non-specific staining was obtained with CLRN1-B2 antibody in human, monkey, rabbit, cat, and rat retinas (data not shown). These data suggest that CLRN1 might be expressed at very low levels in the retina.

Similarly, other published data using CLRN1 antibodies that work well on western blots and on expressed CLRN1 protein *in vitro* do not provide specific localization of the CLRN1 protein on retinal tissue sections by immunocytochemistry [23]. Taken together, these data suggest that antibody access to protein epitopes exposed on the short extracellular and intracellular loops of CLRN1 may be limited. It is known that tetraspanins protrude only 4–5 nm from the plasma membrane [61],[62]. Additionally, tetraspanins are known to function as organizers of multi-molecular membrane complexes [56], similar to USH protein complexes [3],[16],[57],[58]. One explanation for the lack of results by immunocytochemistry on intact tissue (using the antibodies that do stain cultured cells) may be that additional proteins simply block access of antibodies to the CLRN1 epitopes.

### *Clrn1* Knockout Retinas Appear Normal and Cochleas Degenerate

It has been shown that in humans, *CLRN1* mutation results in a phenotype consistent with rod photoreceptor death and RP [10],[59]. However, in mice, histological (Figure 6) and immunohistochemical (Figure 7) analyses in WT (+/+) and *Clrn1* KO (−/−) animals revealed no adverse consequences of *Clrn1* gene deletion. Moreover, ERG analysis of the aged *Clrn1* KO mouse failed to demonstrate either photoreceptor (A-wave) or inner retinal (B-wave) dysfunction (Figure S6) even at 2 years of age. Staining of second and third order neurons (Figure 7A–7H) and the matrix sheaths around cone photoreceptors (Figure 7I and 7J) suggests that these cells are similarly unaffected by *Clrn1* deletion. Thus, structural and functional analyses of *Clrn1* KO retinas reveal that their retinas and their vision is normal.

In contrast, yet similar to other mouse USH KO mouse models, the lack of CLRN1 dramatically affected cochlear structure and function (Figure 8 and Figure S7, respectively). Using ABR in WT C57BL/6J animals as a measure of hearing function, and similar to other data from the *Clrn1* KO mouse [22], we found *Clrn1* KO mice to be essentially deaf as early as postnatal P18 (Figure S7). Whole mount immunostaining of WT (+/+) and *Clrn1* KO (−/−) cochlea show a severe disruption in the stereocilia of 4 month old animals (Figure 8), with some early signs of pathology as early as P15. Thus, in the *Clrn1* KO mouse we have established another model of USH where deafness is apparent, but blindness is absent.

The mechanism underlying the cochlear degeneration remains an open question, and in fact, the role of clarin 1 in the inner ear is still a mystery. Based on homology to Stargazin and its proposed four transmembrane protein structure, CLRN1 was suggested previously to have a role in the excitatory ribbon synapse junctions between hair cells and cochlear ganglion cells [12]. It is interesting to note that *Clrn1* deletion results in a broader distribution of longer stereocilia in cochlear IHCs. While degeneration of hair cells and supporting cells is a common phenomenon in hearing impaired mouse mutants, elongation of stereocilia is usually specific to defects in proteins involved in actin polymerization. The elongated stereocilia present in the *Clrn1* mutant mice suggest a similar role. Stereocilia contain a rigid paracrystalline array of parallel and polarized cross-linked actin filaments, whose length is tightly regulated, with continuous actin turnover [63]. Many factors influence the structure of the actin core of stereocilia, including for example, myosin activity and membrane tension. Defects in actin polymerization and/or depolymerization may be caused by a mutation in any one of the factors that this dynamic process is dependent on, including any of actin's known interacting proteins. For example, myosin VIIa is involved in the dynamic regulation of elongation of stereocilia, as demonstrated by stereociliar defects in *Myo7a* mutant mice [64],[65]. Other genes with mutations leading to Usher syndrome encode proteins that are expressed and are functional in the stereocilia. For example, whirlin, associated with mutations in *USH2A* [18], is expressed in the stereocilia and indeed *Whrn* mouse mutants have shorter stereocilia [66]. Precise localization of clarin1 in the cochlear hair cells and further identification of its interacting proteins [23] will be vital to elucidating the cause for the stereociliar pathology. Our data suggests a new, previously unexplored function for clarin1 in hair cells.

### Conclusions

Similar to most other USH mouse models, our *Clrn1* KO animal exhibits a non-syndromic phenotype. In contrast to our original hypothesis, *Clrn1* is not expressed in photoreceptor cells; but rather, *Clrn1* transcripts are produced by Müller cells, the retinas primary glial cell. These data cannot exclude the possibility of another developmental phase of *Clrn1* expression or that *Clrn1's* expression is absolutely restricted to Müller glia. Additional studies will be required to ascertain how CLRN1 may be participating in the unique relationship between Müller and photoreceptor cells.

Photoreceptors in the *Clrn1* knockout mice remained anatomically and physiologically normal, without exhibiting any phenotypic characteristics of retinal degeneration. In contrast, the *Clrn1* KO mouse is physiologically deaf within 2–3 weeks of birth, although IHCs and OHCs appear largely anatomically intact at this time point. These data suggest that loss of hearing function precedes cell loss in the cochlea: within several months of birth, the absence of CLRN1 protein(s) cause damage evidenced by progressive morphological disruption, proceeding to eventual complete loss of the primary sensory hair cells.

Taken together, these data support the notion that an Usher protein complex [3] is required for inner ear structure and function, but dispensable for retinal structure and function in mice. Moreover, the presence of abnormal hair cell stereocilia in the *Clrn1* KO mouse suggests that CLRN1 may function at the apical part of the cell in addition to (or in place of) serving a role in synapse structure and function, as previously predicted [12]. Our data also suggest that the cellular site of the defect in human sensory tissues must be ascertained to design a rational therapy for these debilitating conditions, and early diagnosis and intervention will be critical to the success of human therapies for USH3-related hearing and vision losses.

## Materials and Methods

### Knockout Mouse Production

A *Clrn1* knockout mouse was generated by IngenKO, Pty. Ltd. (Clayton, Victoria, Australia). Briefly, the targeting construct was produced using the ET cloning system [67] with C57BL/6J genomic DNA. The construct included loxP sites that flanked part of the *Clrn1* upstream promoter, the first exon (5′ UTR and protein coding sequence), 269 bp of the first intron, and a neomycin resistance gene (NeoR). The targeting construct was electroporated into C57BL/6J embryonic stem (ES) cells, and recombinant clones were selected using G418. Selected clones were transfected with Cre recombinase and screened by PCR for removal of the *Clrn1* gene fragment noted above. Recombined ES cells were microinjected into BALB/c blastocysts and implanted into pseudo-pregnant mothers. Chimeric progeny were obtained, and highly chimeric animals were subsequently mated with C57BL/6J mice to identify germ-line transmission of the *Clrn1* deletion. Founder animals were identified, and heterozygous progeny were delivered to University of California, Berkeley. Heterozygous animals were bred to generate homozygous *Clrn1* KO animals. WT C57BL/6J control, *Clrn1* heterozygous (+/−), and *Clrn1* homozygous (−/−) mice were maintained up to 2 years for histological, histochemical, electrophysiological, and biochemical analyses. All experiments were performed in adherence to the ARVO Statement for the Use of Animals in Ophthalmic and Vision Research, and were also approved by the Animals Care and Use Committee of the University of California, Berkeley.

### Nucleic Acid Isolation and Reverse Transcription

Total RNA was isolated from numerous tissue sources, including WT adult C57BL/6J mice, *Clrn1* KO (−/−,+/−) mice, C3H/HeJ (C3H) mice, WT Sprague-Dawley (SD) rats, P23H-3 (rhodopsin mutant) transgenic rats, and Royal College of Surgeons (RCS) rats. For adult retinas and brain regions, tissues samples were carefully dissected from any surrounding tissue. For fetal tissues, timed pregnancies were used, and whole fetuses, heads, brains, eyes, or retinas were dissected. Tissues were immediately homogenized in a 1.5 mL microfuge tube containing 100–300 µL of Tri-Reagent (Sigma-Aldrich) using a small plastic sterile pestle (Fisher Scientific, Pittsburgh, PA). Total RNA was extracted, precipitated, and resuspended in ddH_2_O according to manufacturer's instructions. For cultured Müller cell studies (a gift from Vijay Sarthy), 6-well or 10 cm plates of cells were grown to near confluence, and total RNA was isolated using an RNeasy kit (Qiagen Corp., Valencia, CA) per the manufacturer's instructions. Purity was assessed and total RNA was quantified with 260 and 280 nm light. RNA was reverse transcribed using M-MLV reverse transcriptase (Promega) according to manufacturer's instructions. A standard reverse transcription reaction was performed for 2 hours at 37°C, and included 25–50 ng/µL input total RNA. After the 2 hour transcription reaction, samples were stored at −20°C until used for PCR reactions.

### Reverse Transcription and Polymerase Chain Reaction

All oligonucleotide DNA primers (Figure S1) were ordered from Operon Technologies (Huntsville, AL). Multiple mouse-specific primer pairs were designed to elucidate *Clrn1* splice variant expression, developmental expression, and tissue distribution. Total RNA samples were reverse transcribed using M-MLV reverse transcriptase. Twenty-five to 50 ng of cDNA was used as template in all reactions, and all were amplified for 30 cycles, utilizing a 65°C annealing temperature. Polymerase chain reaction (PCR) was performed according to standard thermocycling conditions, using 2.5 mM Mg++, 200 nM primers, 0.1 U Taq Polymerase, 200 nM dNTPs, and 1X PCR buffer. Cycling conditions were typically 60°C for 30” (annealing), 72°C for 1′ (extension), and 94°C for 20” (melting) for 30–35 cycles. Reaction products were electrophoresed through 1–2% agarose gels containing 100 ng/mL ethidium bromide, buffered by Tris-Acetate, pH 8.0, and photographed on a Kodak EDAS 290 imaging system (Fisher Scientific).

### Real-Time Quantitative RT–PCR

Quantitative real-time RT-PCR was used to compare expression of the two primary splice variants, and for quantitative gene expression in the C3H/HeJ (C3H) mouse retina. For splice variant analysis, FAM/BHQ probes (Biosearch Technologies, Novato, CA) were designed to span exon splice junctions (probes crossed the exon 1/3 or exon 1/4 junctions) in order to distinguish between the highly similar transcripts. Expression was normalized to 28S ribosomal RNA for all 6 WT mice. For quantitative gene expression in animal models of retinal degeneration (C3H mice, RCS and P23H-3 rats), SYBR green was the reporter molecule used to assess expression of several retinal transcripts: *cFos, Slc1a3, Grm1, Rhod, Rp-1, Thy1, Clrn1*, and *Vim*. Two eyes for each strain/genotype were assayed, each measured in duplicate, and the average expression levels are shown. Amplification, detection, and quantification were performed on an Mx3000P real-time thermocycler (Stratagene Corp., La Jolla, CA). Refer to Figure S1 for primers used. Twenty-five or 50 ng of cDNA was included in each reaction, along with 300 nM primers (final concentration) and 2X Power SYBR Green PCR Master Mix (Applied Biosystems, Foster City, CA). The reactions were cycled 45 times with a final melting curve analysis at the end of the experiments to qualify primer specificity. All primer pairs showed single peaks in the melting curve analysis (data not shown).

### In Situ Hybridization

Animals were housed in the Department of Comparative Medicine at the University of Washington and were euthanized according to approved protocols. Timed pregnant female mice were sacrificed and embryos removed at E18.5. The embryos were fixed in a modified Carnoy's solution (60% ethanol∶4% formaldehyde:10% acetic acid) overnight at 4°C. The samples were washed and dehydrated in 100% ethanol overnight at 4°C, and then were embedded in paraffin and 6 micrometer sections were collected. For the postnatal ages, we noted the day of birth as P0 and sacrificed pups at P0 and P3 according to approved protocols. We then dissected the retinas from the animals, and fixed them and processed them for paraffin sectioning as described above. At least 3 animals were examined at each time point. Mouse *Clrn1* cDNA (clone ID: 40130533) to exons 1 and 4, including 154 bp and 646 bp of 5′ and 3′ UTR sequence was obtained from Open Biosystems Inc. Digoxigenin (DIG)-labeled probes were prepared according to the manufacturer's manual for DIG-11-UTP (Roche, Indianapolis, IN) and the hybridization was carried out according to Hayashi et al. [68]. The in situ product was visualized using anti-DIG alkaline phosphatase conjugated secondary antibody (Roche) and NBT/BCIP liquid substrate system (Sigma, St. Louis, MO). After ISH, the slides were fixed with 4% paraformaldehyde for 1 hour and washed in PBS. The slides were then incubated with 10% fetal bovine serum and 2% nonfat dry milk in PBS/0.1% Triton X-100 (PBST) for 30 minutes. After an overnight incubation with the primary rabbit antibody (anti-Glast, #AB1782, Millipore Corp., Billerica, MA) at 1∶200 dilution, or rabbit anti-beta-tubulin III (TuJ1, Covance, Austin, TX) at 1∶300 dilution, the slides were washed and incubated in fluorescent conjugated secondary antibody, rinsed with PBST and mounted under a cover glass with Fluoromount G (Southern Biotechnology, Birmingham, AL). Images were captured with a Zeiss Axioplan microscope using a SPOT CCD camera (Diagnostic Instruments, Sterling Heights, MI) and similarly processed using Adobe Photoshop (Adobe Systems, San Jose, CA).

### Laser Capture Microdissection

LCM was used to isolate total RNA from the three nuclear layers of the retina: outer nuclear layer (ONL), INL, and ganglion cell layer (GCL). Briefly, whole eyes were enucleated and immediately frozen in tissue freezing medium in a dry ice/ethanol slurry. Seven µm retinal sections were collected onto ProbeON Plus microscope slides (Fisher Scientific), and stored in 70% ethanol at -25°C. Slides were dehydrated at RT in graded ethanol series (80%, 90%, 3×100%) and 2 changes of 100% xylenes, then air dried in a fume hood, and immediately processed with a PixCell II laser capture system (Arcturus, Mountain View, CA). Laser capture was performed as previously described [69]. Total RNA was isolated using a Pico Pure extraction kit (Arcturus) according to manufacturer's protocol. The optional DNAse step was omitted, and primers that span introns were used for analysis. Two rounds of amplification were used to increase sensitivity while mitigating background amplification. For *Rp1*, the F1/R1 primer pair was used for both rounds of amplification, whereas for *Clrn1*, F2/R2 primers were used for the first round, and F1/R1 primers were used for the second round of amplification (see Figure S1). The second round of amplification used 1/100^th^ of the first round PCR product as template for the second round of PCR. Reverse transcription was performed as described above.

### Retinal Histology

Eyes for histological analysis were processed as previously described [70]. Eyes were isolated from 9 month old littermate *Clrn1* KO and age-matched WT C57BL/6J animals after cardiac perfusion with 2.5% formaldehyde/2% glutaraldehyde in PBS (pH 7.4). Eyes were immersion fixed for at least 24 hours. The samples were dissected, osmicated, washed, and dehydrated in a graded ethanol and embedded in Epon-Araldite resin (Ted Pella, Inc., Redding, CA). Following polymerization, 1 µm sections were cut on a microtome (UltraCut; Leica, Deerfield, IL) and stained with 1% toluidine blue. Images were digitally captured with a CCD camera (Axiocam; Carl Zeiss Meditec, Inc., Thornwood, NY) on an Axiophot II photomicroscope (Carl Zeiss, Oberkochen, Germany).

### Clarin 1 Antibody Production

Several antibody production technologies were used to generate reagents for immunological analyses. We generated two rabbit antibodies (Sigma-Genosys/Sigma-Aldrich, St. Louis, MO) to two human CLRN1 peptides (5′-ASGQELDKFMGEM(C)-3′; aa (amino acids) 49–61; “CLRN1-A”) and (5′-(C)GFQFPFAKSKDAE-3′; aa 209–222; “CLRN1-B”). New Zealand White rabbits were injected with peptides conjugated to keyhole limpet hemocyanin (KLH). Serum from the B-peptide showed high specificity in vitro, and CLRN1-B2 antibodies were affinity purified and used in immunohistochemistry, western blotting, and IP experiments. We also generated chicken IgY antibodies by immunizing chicken eggs with two mouse peptides (5′-KVHRLSEKIANFKE-3′; aa 160-173; “Clarin160”) and (5′-PFTKSKETETTNVA-3′; aa 214-227; “Clarin214”). Both antibodies were affinity purified and tested in transfected HEK293 cells, western blotting, and immunohistochemistry.

### Immunocytochemistry

Standard immunocytochemical procedures were followed. Procedures were performed at room temperature (RT) unless indicated otherwise. Briefly, after fixation, the cells were rinsed once in phosphate buffered saline (PBS, pH 7.4) and twice with PBT (PBS including 0.1% Triton X-100 detergent (Sigma-Aldrich) and 0.1% bovine serum albumin (BSA; Sigma-Aldrich)). Antibody dilution buffer (ADB) was made by adding 2% BSA and 1∶100 normal donkey serum (Jackson ImmunoResearch Laboratories, Inc., West Grove, PA) to PBT. The cells were blocked for 30 minutes in ADB, then primary antibodies diluted in ADB were added following removal of the blocking buffer. After one hour, the antibodies were removed and the cells were rinsed with PBT 3×10′. Finally, Cy3 conjugated secondary antibodies (Jackson ImmunoResearch) diluted 1∶500 in ADB were added to the cells for 30′. After rinsing again 3×10′ with PBT and 2×5′ with PBS, the cover glass with cells attached was inverted onto a clean microscope slide containing a drop of Vectashield hard set mounting medium including DAPI (Vector Laboratories, Inc., Burlingame, CA). Cells were photographed on an Axiophot II photomicroscope (Carl Zeiss) fitted with an AxioCam digital camera and fluorescence filters for three color emission. Images were identically post-processed using Adobe Photoshop (Adobe Systems).

### Cryosection Tissue Processing and Immunohistochemistry

WT and *Clrn1* KO mouse eyes were isolated following euthanasia by CO_2_ overdose. Eyes were removed rapidly and fixed in 10% neutral buffered formalin (NBF; Ted Pella, Redding, CA) for at least 24 hours, rotating at 4°C. Eyes were opened and the cornea, lens, and vitreous were removed, leaving the posterior pole containing the retina. The posterior pole was rinsed in PBS and equilibrated into 30% sucrose (in PBS) overnight. Eyecups were embedded in Tissue Freezing Medium (TFS; TBS, Durham, NC), and 12–16 µm thick sections were mounted on probe-on plus microscope slides and air-dried. For histology, the tissue sections were counterstained with hematoxylin. For cryosection immunohistochemical procedures, we utilized essentially the same reagents and concentrations as for immunocytochemistry, except that incubation times were increased due to the diffusion of reagents into and out of the thicker tissue samples. For CLRN1-B2 immunohistochemistry (Figure S5), 12 µm thick cryosections were used. Briefly, sections were thawed, blocked with 5% normal donkey serum in PBT and incubated with primary antibodies diluted in PBT overnight at 4°C. Sections were rinsed and incubated in secondary antibodies (anti-rabbit Cy3), rinsed again and visualized on a Zeiss Axiophot 2 confocal microscope (Carl Zeiss).

### Thick-Section Confocal Microscopy

Tissue processing for confocal microscopy was performed as described previously [71]. Briefly, eyes and subsequently anterior eye structures were removed, and posterior poles were immersion fixed in 10% NBF for at least 2 hours. Whole retinas were gently removed from the eyecups following severing of the optic nerve head. Retinas were rinsed well with PBS, and embedded in low-melting temperature agarose (5%) at ∼40°C. After cooling, the agarose blocks were sectioned on a Leica vibratome at 100 µm, and collected in PBS. Sections were selected and placed into antibody dilution buffer (ADBX (TNTB; 100 mM Tris (pH 7.4), 150 mM NaCl, 0.1% BSA, 0.1% Triton X-100) plus 1% BSA and 1% normal donkey serum). Sections were incubated for at least 16 hours at 4°C with gentle rocking. The following day, the blocking solution was removed and primary antibodies diluted in ADBX were added for another ∼16–24 hour incubation, rocking at 4°C. Several primary antibodies were used, including: the lectin peanut agglutinin (PNA)-Rhodamine (Sigma-Aldrich, St. Louis, MO), anti-synaptophysin (mouse monoclonal, 1∶500, DAKO North America Inc., Carpinteria, CA), anti-vimentin (mouse monoclonal, 1∶500, DAKO), anti-GFAP (rabbit polyclonal, 1∶500, DAKO), anti-PKCalpha (rabbit polyclonal, 1∶250, Biomol, Plymouth Meeting, PA), anti-Calbindin (rabbit polyclonal ab11426, 1∶250, Abcam Inc., Cambridge, MA), and custom anti-CLRN1 antibodies (see above) generated in rabbits and chickens (1∶100–1∶500). The following morning, the primary antibodies were removed, and the tissue sections were rinsed briefly with PBS, 3×15′ in TNTB, and once for 2 hours in TNTB (rocking during each rinse). Following the rinsing, sections were incubated for ∼16 hours in Cy3 conjugated secondary antibodies diluted in ADBX. Sections were again rinsed as above, followed by an additional ddH_2_O rinse. Sections were then counterstained with SYTOX Green (Invitrogen) diluted 1∶1000 in ddH_2_O for 15′, rocking at 4°C. The sections were rinsed with ddH2O, then with PBS for 15′, and mounted under a cover glass using hard set Vectashield (Vector Laboratories) for fluorescence without DAPI. Cover glass was sealed with nail polish to prevent evaporation. Sections were imaged on a Zeiss Axiophot 2 confocal microscope (Carl Zeiss), and comparison images were identically post-processed using Adobe Photoshop (Adobe Systems).

### Western Blotting and Immunoprecipitation

Triton X-100-soluble and SDS-soluble proteins from adult donor human, mouse, and rat retinas were isolated following whole retinal homogenization, and quantified by BCA assay. Twenty micrograms of total protein was boiled and loaded per lane, electrophoresed, and transferred to a nylon membrane. The membrane was blocked with 2% BSA in PBT, incubated in primary antibody (CLRN1-B2) at 1∶3000 (0.4 µg/mL) overnight at RT, rinsed with PBT, and incubated in anti-rabbit alkaline phosphatase-conjugated secondary antibody at 1∶2000 for 1 hour at RT. Following final rinsing, the blot was incubated in NBT/BCIP (Roche) to produce a blue precipitate. For IP experiments, we used the Seize X IP kit (Pierce Biotechnology, Rockford, IL). The CLRN1-B2 antibody was conjugated to agarose beads by cross linking with cyanogen bromide according to the manufacturer's protocol. Approximately 200 µg of total retinal protein (from 4 combined adult mouse retinas) was incubated overnight at 4°C with the agarose bead-bound antibodies. The beads were washed several times, and the immunoprecipitated proteins were eluted 3 times. Ten per cent of each eluate was loaded on each lane of a polyacrylamide gel and processed for standard western blotting (see above).

### Electroretinography

Electroretinography was performed using standard procedures. Briefly, mice and rats were dark adapted overnight, anesthetized with xylazine (13 mg/ml) and ketamine (87 mg/ml) under dim red light illumination, and maintained on a heating pad. Corneas were locally anesthetized with 0.5% proparacaine, and pupils were dilated with 2.5% phenylephrine and 1% atropine sulfate drops. Full field scotopic electroretinograms (ERGs) were acquired following 10 µs flashes of white light delivered by an Espion Colorburst mini-Ganzfeld stimulator (Diagnosys LLC, Littleton, MA). Retinal responses were recorded using gold wire corneal contact lens electrodes. A 6-step stimulation protocol was used with intensities ranging from -4 log candela second per square meter (cd.s/m^2^) to 1 log cd.s/m^2^. Three responses were recorded and averaged for each stimulus intensity level. A-wave amplitude was measured from the baseline to the first negative peak. B-wave amplitude was measured from the A-wave negative peak to the most positive peak. WT (−/−) and *Clrn1* KO mice aged up to 24 months were subjected to ERG analysis. At least six animals were recorded for each genotype at each age. Data were analyzed using Student's *t*-test. Recording traces were averaged for each light intensity level, and error bars represent one standard deviation.

### Cochlear Immunohistochemistry

For whole mount immunohistochemistry, samples were fixed in 4% PFA overnight at 4°C and then washed with PBS. Auditory (from middle and apical turn of cochlea) and vestibular sensory epithelia were isolated and then incubated with 0.05% Triton in 10% normal donkey serum (NDS) for 1 hour at RT. Tissues were incubated overnight with rabbit anti-myosin VI primary antibody (Proteus Biosciences, Ramona, CA) at a dilution of 1∶200 in a green buffer (Dyna Scientific, Logan, UT). The tissues were then washed and incubated for 2 hours at RT with a secondary antibody conjugated to AlexaFlour488 and rhodamine-conjugated Phalloidin (Invitrogen - Molecular Probes, Carlsbad, CA), and after final rinsing were mounted with fluorescent mounting medium (GBI-Golden Bridge International, Mukilteo, WA). All images were acquired on a Leica Confocal Microscope TCS-SP5.

### Cochlear Quantitative Measurement Analysis

The length of the stereocilia from the tallest row of the inner hair cells bundle was measured using Image J (http://rsbweb.nih.gov/ij/) and used for quantitative analyses. A minimum of 8 images of inner hair cells were acquired from the mid-apex turn of the cochlea (60–80% of the cochlear length) from three different animals of each genotype. All images were taken with the same magnification and exposure parameters. *t*-tests were performed using SPSS software.

### Auditory Brainstem Response

Brainstem responses were recorded for *Clrn1* KO and WT C57BL/6J mice between P16 and P42. Following deep anesthesia using ketamine and xylazine, animals were maintained on a warming pad, and three needle electrodes (recording, reference, and ground) were inserted subcutaneously. Tone pips (3 ms duration, frequencies at 8, 16, or 32 kilohertz (kHz) and intensities at 40, 60, or 80 dB SPL (decibel sound pressure level)) were delivered to the left ears at a rate of 19 times per second through a calibrated earphone (Stax Ltd., Japan). ABR signals were recorded using BioSigRP software on a Tucker Davis Technology System 3 recording rig (Alachua, FL), and 500 recordings were averaged for each animal in each condition.

### EGFP-CLRN1 Fusion Construct and Transfection

The pEGFP-C1 vector (GI:1377914; Clontech, Mountain View, CA) was utilized for generating a fusion construct between EGFP and CLRN1. The full-length mouse *Clrn1* coding sequence (exons 1, 3, and 4, and including a stop codon; 699 bp) was cloned from C57BL/6J genomic DNA. The clone was sequenced and spliced into the *Bam*HI and *Eco*RI restriction sites within the pEGFP-C1 multiple cloning site at the 3′ end of, and in frame with, EGFP. Viable fusion protein expression was evidenced by a redistribution of fluorescence from the cytoplasm to the plasma membrane in transfection assays (see Figure S4). HEK293 cells were used for all transfection assays. Transfection with the EGFP-CLRN1 construct was performed essentially as described previously [72]. Briefly, plasmid DNA was mixed with Lipofectamine 2000 (Invitrogen) and incubated with ∼50% confluent HEK293 cells grown on cover glass. After 24–36 hours, the cells were fixed in 10% neutral buffered formalin for 15′, and subjected to immunocytochemical analysis.

## Supporting Information

Figure S1 Oligonucleotide primers and fluorescent probes used.
(0.40 MB PDF)

Figure S2 *Clrn1* mRNA co-localizes with a marker for Müller cells. (A–C) *Clrn1* ISH combined with anti-Glast (Slc1a3; a Müller glial marker) immunohistochemistry labeling shows co-labeling. The labeling in the GCL represents Müller cell processes, which extend to and surround cells in the GCL. (D–F) Higher magnification view of the region in the box in H, and the arrow points to a cluster of cells that is labeled with both *Clrn1* and Glast. Scale bars = 50 µm.
(1.42 MB TIF)

Figure S3 Genetically Degenerated rat retinas express *Clrn1* despite photoreceptor loss. (A,B) Photomicrographs of retinal cross-sections from a two month old WT Sprague-Dawley rat (A) and a 1 year old P23H-3 (P23H-3; rhodopsin mutant) transgenic rat (B). Note the apposition of the RPE and INL in the P23H retina, and thus, a complete absence of the outer nuclear layer (ONL). Tissues were stained with hematoxylin. (C) Using RT-PCR, both SD and P23H rats (duplicate samples for each) express *Clrn1*. In contrast, only the WT animal expresses *Rp1*, supporting the histological loss of photoreceptors in the P23H animals. (D) Similar results are observed in the RCS rat. The congenic control strain (rdy+) expresses both *Clrn1* and *Rp1* by RT-PCR, whereas the RCS mutant animals express *Clrn1*, but not *Rp1*. (E) Quantitative RT-PCR analysis in the two rat models of photoreceptor degeneration indicates ∼100-fold reductions in the expression of *Rp1*. Due to equal amounts of retinal cDNA being used in each reaction, a relative increase in *Clrn1* expression is observed in mutant animals due to the loss of photoreceptor mRNA in the total retinal RNA pool used for cDNA synthesis. Data represent the averages of two animals in each condition. Scale bar = 50 µm.
(1.04 MB TIF)

Figure S4 Clarin 1 localizes to the plasma membrane in transiently transfected HEK293 cells, and anti-CLRN1 antibodies specifically detect CLRN1. Clarin 1 Localizes to the Plasma Membrane in Transiently Transfected HEK293 Cells, and Anti-CLRN1 Antibodies Specifically Detect CLRN1. (A) Transfection with the pEGFP-C1 parent plasmid alone results in bright green cytoplasmic fluorescence in HEK293 cells. (B) The mouse *Clrn1* cDNA (exons 1, 3, and 4) was cloned in-frame with EGFP. Following transfection, the EGFP-CLRN1 fusion protein caused the redistribution of EGFP (green fluorescence) from the cytoplasm to the plasma membrane. (C–E) CLRN1-B2 antibody staining of HEK293 cells transfected with the EGFP-CLRN1 fusion protein. The merged image (E) shows near perfect co-localization (yellow). Cells were counterstained with DAPI. (F–H) HEK293 cells were transfected with the EGFP-CLRN1 fusion protein construct and immunostained with a different anti-CLRN1 antibody (Clarin160). (I–K) Cells were transfected with the EGFP-CLRN1 fusion protein construct and immunostained with secondary antibodies alone. Only green fluorescence originating from EGFP is visible. (L) Western blot using the CLRN1-B2 antibody on human (H), mouse (M), and rat (R) retinal homogenates, with and without SDS in the sample buffer, including BME. The CLRN1-B2 antibody detects a ∼26 kDa band in mouse and rat, and a doublet of ∼25 kDa and ∼34 kDa in the human retinal sample.
(2.01 MB TIF)

Figure S5 Immunostaining of P7 WT and *Clrn1* KO mouse retinal sections. (A–H) Similar staining patterns were observed in WT and *Clrn1* KO P7 retinal tissues immunostained with GFAP (A–B), vimentin (C–D), Clarin160 (E–F), and CLRN1-B2 antibodies. Though there may be a slight reduction in labeling in the *Clrn1* KO mouse (F and H), non-specific (background) staining in the tissues prevents reliable use of either anti-CLRN1 antibody to specifically localize CLRN1 in the retina. Scale bar = 20 µm. ONL = outer nuclear layer, INL = inner nuclear layer, and GCL = ganglion cell layer.
(2.74 MB TIF)

Figure S6 Electroretinograms of WT and *Clrn1* KO (−/−) mice at 18 months of Age. (A,B) White traces represent WT C57BL/6J mice, while black traces represent *Clrn1* KO (−/−) mice. (A) Peak A-wave voltage (µV) responses to increasing light intensities (log cd.s/m2), from left to right. (B) Peak B-wave voltage responses to increasing light intensities, from left to right. Both A- and B- waves are similar for the WT and *Clrn1* KO (−/−) animals. (C) Graph illustrating voltage changes vs. time for both the WT and *Clrn1* KO (−/−) animals. Error bars = 1 SD.
(0.13 MB TIF)

Figure S7 Auditory brainstem response recordings of inner ear function. (A) Ten millisecond (ms) auditory brainstem responses (ABR) in WT C57BL/6J and *Clrn1* KO (−/−) mice at 6 weeks of age. Averaged traces are shown for five animals in each condition, exposed to 3 sound intensities (40, 60, and 80 dB SPL) at three frequencies (8, 16, and 32 kHz). *Clrn1* KO animals exhibit a profound diminution in their brainstem responses at all the dB SPL levels tested, and tonal frequency had no effect on the ABR. (B) Ten ms 80 dB SPL Auditory Brainstem Response (ABR) recordings from WT C57BL/6J (P16) and *Clrn1* KO (P18) mice. Five animals were recorded for each genotype; individual traces are shown on the left, and averaged curves are shown on the right. Even at this early age, *Clrn1* KO animals show severely depressed ABR sensitivities. A 1 ms bar is shown for reference.
(0.17 MB TIF)

Figure S8 P120 vestibular hair cells of *Clrn1* KO mice appear unaffected. (A–D) Immunofluorescent confocal images of inner ear sensory maculae harvested from utricles. Samples were labeled with rhodamine-Phalloidin and are shown at low (A and B) and high (C and D) magnification. *Clrn1* KO mice show a grossly normal utricular elliptical shape (B) with classical distribution of hair cells (D). Scale bars: In A (200 µm) applies to A and B; in C (20 µm) applies to C and D.
(0.33 MB TIF)

Video S1 An Aged Homozygous *Clrn1* KO (−/−) Mutant Mouse Displaying Circling Behavior.
(3.78 MB MOV)

## References

[1] Vernon M (1969). Usher's syndrome–deafness and progressive blindness. Clinical cases, prevention, theory and literature survey.. J Chronic Dis.

[2] Petit C (2001). Usher syndrome: from genetics to pathogenesis.. Annu Rev Genomics Hum Genet.

[3] Reiners J, Nagel-Wolfrum K, Jurgens K, Marker T, Wolfrum U (2006). Molecular basis of human Usher syndrome: deciphering the meshes of the Usher protein network provides insights into the pathomechanisms of the Usher disease.. Exp Eye Res.

[4] Saihan Z, Webster AR, Luxon L, Bitner-Glindzicz M (2009). Update on Usher syndrome.. Curr Opin Neurol.

[5] Pennings RJ, Fields RR, Huygen PL, Deutman AF, Kimberling WJ (2003). Usher syndrome type III can mimic other types of Usher syndrome.. Ann Otol Rhinol Laryngol.

[6] Aller E, Jaijo T, Oltra S, Alio J, Galan F (2004). Mutation screening of USH3 gene (clarin-1) in Spanish patients with Usher syndrome: low prevalence and phenotypic variability.. Clin Genet.

[7] Otterstedde CR, Spandau U, Blankenagel A, Kimberling WJ, Reisser C (2001). A new clinical classification for Usher's syndrome based on a new subtype of Usher's syndrome type I.. Laryngoscope.

[8] Smith RJ, Berlin CI, Hejtmancik JF, Keats BJ, Kimberling WJ (1994). Clinical diagnosis of the Usher syndromes. Usher Syndrome Consortium.. Am J Med Genet.

[9] Pakarinen L, Tuppurainen K, Laippala P, Mantyjarvi M, Puhakka H (1995). The ophthalmological course of Usher syndrome type III.. Int Ophthalmol.

[10] Herrera W, Aleman TS, Cideciyan AV, Roman AJ, Banin E (2008). Retinal Disease in Usher Syndrome III Caused by Mutations in the Clarin-1 Gene.. Invest Ophthalmol Vis Sci.

[11] Plantinga RF, Pennings RJ, Huygen PL, Sankila EM, Tuppurainen K (2006). Visual impairment in Finnish Usher syndrome type III.. Acta Ophthalmol Scand.

[12] Adato A, Vreugde S, Joensuu T, Avidan N, Hamalainen R (2002). USH3A transcripts encode clarin-1, a four-transmembrane-domain protein with a possible role in sensory synapses.. Eur J Hum Genet.

[13] Fields RR, Zhou G, Huang D, Davis JR, Moller C (2002). Usher syndrome type III: revised genomic structure of the USH3 gene and identification of novel mutations.. Am J Hum Genet.

[14] Joensuu T, Hamalainen R, Yuan B, Johnson C, Tegelberg S (2001). Mutations in a novel gene with transmembrane domains underlie Usher syndrome type 3.. Am J Hum Genet.

[15] Sankila EM, Pakarinen L, Kaariainen H, Aittomaki K, Karjalainen S (1995). Assignment of an Usher syndrome type III (USH3) gene to chromosome 3q.. Hum Mol Genet.

[16] Kremer H, van Wijk E, Marker T, Wolfrum U, Roepman R (2006). Usher syndrome: molecular links of pathogenesis, proteins and pathways.. Hum Mol Genet.

[17] Ness SL, Ben-Yosef T, Bar-Lev A, Madeo AC, Brewer CC (2003). Genetic homogeneity and phenotypic variability among Ashkenazi Jews with Usher syndrome type III.. J Med Genet.

[18] Ebermann I, Scholl HP, Charbel Issa P, Becirovic E, Lamprecht J (2007). A novel gene for Usher syndrome type 2: mutations in the long isoform of whirlin are associated with retinitis pigmentosa and sensorineural hearing loss.. Hum Genet.

[19] Sadeghi M, Cohn ES, Kimberling WJ, Tranebjaerg L, Moller C (2005). Audiological and vestibular features in affected subjects with USH3: a genotype/phenotype correlation.. Int J Audiol.

[20] Williams DS (2008). Usher syndrome: animal models, retinal function of Usher proteins, and prospects for gene therapy.. Vision Res.

[21] Liu X, Bulgakov OV, Darrow KN, Pawlyk B, Adamian M (2007). Usherin is required for maintenance of retinal photoreceptors and normal development of cochlear hair cells.. Proc Natl Acad Sci U S A.

[22] Geng R, Geller SF, Hayashi T, Ray CA, Reh TA (2009). Usher Syndrome IIIA Gene Clarin-1 is Essential for Hair Cell Function and Associated Neural Activation.. Hum Mol Genet.

[23] Tian G, Zhou Y, Hajkova D, Miyagi M, Dinculescu A (2009). Clarin-1, encoded by the Usher syndrome III causative gene, forms a membranous microdomain: Possible role of Clarin-1 in organizing the actin cytoskeleton.. J Biol Chem.

[24] Zallocchi M, Meehan DT, Delimont D, Askew C, Garrige S (2009). Localization and expression of clarin-1, the Clrn1 gene product, in auditory hair cells and photoreceptors.. Hear Res.

[25] Poche RA, Furuta Y, Chaboissier MC, Schedl A, Behringer RR (2008). Sox9 is expressed in mouse multipotent retinal progenitor cells and functions in Muller glial cell development.. J Comp Neurol.

[26] Bowes C, Li T, Danciger M, Baxter LC, Applebury ML (1990). Retinal degeneration in the rd mouse is caused by a defect in the beta subunit of rod cGMP-phosphodiesterase.. Nature.

[27] Carter-Dawson LD, LaVail MM, Sidman RL (1978). Differential effect of the rd mutation on rods and cones in the mouse retina.. Invest Ophthalmol Vis Sci.

[28] Chang B, Hawes NL, Hurd RE, Davisson MT, Nusinowitz S (2002). Retinal degeneration mutants in the mouse.. Vision Res.

[29] Lewis GP, Guerin CJ, Anderson DH, Matsumoto B, Fisher SK (1994). Rapid changes in the expression of glial cell proteins caused by experimental retinal detachment.. American Journal of Ophthalmology.

[30] Lewin AS, Drenser KA, Hauswirth WW, Nishikawa S, Yasumura D (1998). Ribozyme rescue of photoreceptor cells in a transgenic rat model of autosomal dominant retinitis pigmentosa.. Nat Med.

[31] LaVail MM (2001). Legacy of the RCS rat: impact of a seminal study on retinal cell biology and retinal degenerative diseases.. Prog Brain Res.

[32] Blackshaw S, Fraioli RE, Furukawa T, Cepko CL (2001). Comprehensive analysis of photoreceptor gene expression and the identification of candidate retinal disease genes.. Cell.

[33] Redmond TM, Yu S, Lee E, Bok D, Hamasaki D (1998). Rpe65 is necessary for production of 11-cis-vitamin A in the retinal visual cycle.. Nat Genet.

[34] Boughman JA, Vernon M, Shaver KA (1983). Usher syndrome: definition and estimate of prevalence from two high-risk populations.. J Chronic Dis.

[35] Tuson M, Marfany G, Gonzalez-Duarte R (2004). Mutation of CERKL, a novel human ceramide kinase gene, causes autosomal recessive retinitis pigmentosa (RP26).. Am J Hum Genet.

[36] Berson EL, Grimsby JL, Adams SM, McGee TL, Sweklo E (2001). Clinical features and mutations in patients with dominant retinitis pigmentosa-1 (RP1).. Investigative Ophthalmology & Visual Science.

[37] Humphries P, Kenna P, Farrar GJ (1992). On the molecular genetics of retinitis pigmentosa.. Science.

[38] Hamel C (2006). Retinitis pigmentosa.. Orphanet J Rare Dis.

[39] Hamel CP (2007). Cone rod dystrophies.. Orphanet J Rare Dis.

[40] Lem J, Krasnoperova NV, Calvert PD, Kosaras B, Cameron DA (1999). Morphological, physiological, and biochemical changes in rhodopsin knockout mice.. Proc Natl Acad Sci U S A.

[41] Pittler SJ, Baehr W (1991). Identification of a nonsense mutation in the rod photoreceptor cGMP phosphodiesterase beta-subunit gene of the rd mouse.. Proc Natl Acad Sci U S A.

[42] Kedzierski W, Nusinowitz S, Birch D, Clarke G, McInnes RR (2001). Deficiency of rds/peripherin causes photoreceptor death in mouse models of digenic and dominant retinitis pigmentosa.. Proc Natl Acad Sci U S A.

[43] Clarke G, Goldberg AF, Vidgen D, Collins L, Ploder L (2000). Rom-1 is required for rod photoreceptor viability and the regulation of disk morphogenesis.. Nat Genet.

[44] Furukawa T, Morrow EM, Li T, Davis FC, Cepko CL (1999). Retinopathy and attenuated circadian entrainment in Crx-deficient mice.. Nat Genet.

[45] Ohlemiller KK, Hughes RM, Lett JM, Ogilvie JM, Speck JD (1997). Progression of cochlear and retinal degeneration in the tubby (rd5) mouse.. Audiol Neurootol.

[46] Yang RB, Robinson SW, Xiong WH, Yau KW, Birch DG (1999). Disruption of a retinal guanylyl cyclase gene leads to cone-specific dystrophy and paradoxical rod behavior.. J Neurosci.

[47] Pang JJ, Chang B, Hawes NL, Hurd RE, Davisson MT (2005). Retinal degeneration 12 (rd12): a new, spontaneously arising mouse model for human Leber congenital amaurosis (LCA).. Mol Vis.

[48] Alagramam KN, Murcia CL, Kwon HY, Pawlowski KS, Wright CG (2001). The mouse Ames waltzer hearing-loss mutant is caused by mutation of Pcdh15, a novel protocadherin gene.. Nat Genet.

[49] Di Palma F, Holme RH, Bryda EC, Belyantseva IA, Pellegrino R (2001). Mutations in Cdh23, encoding a new type of cadherin, cause stereocilia disorganization in waltzer, the mouse model for Usher syndrome type 1D.. Nat Genet.

[50] Gibson F, Walsh J, Mburu P, Varela A, Brown KA (1995). A type VII myosin encoded by the mouse deafness gene shaker-1.. Nature.

[51] Johnson KR, Gagnon LH, Webb LS, Peters LL, Hawes NL (2003). Mouse models of USH1C and DFNB18: phenotypic and molecular analyses of two new spontaneous mutations of the Ush1c gene.. Hum Mol Genet.

[52] Kikkawa Y, Shitara H, Wakana S, Kohara Y, Takada T (2003). Mutations in a new scaffold protein Sans cause deafness in Jackson shaker mice.. Hum Mol Genet.

[53] Haywood-Watson RJ, Ahmed ZM, Kjellstrom S, Bush RA, Takada Y (2006). Ames Waltzer deaf mice have reduced electroretinogram amplitudes and complex alternative splicing of Pcdh15 transcripts.. Invest Ophthalmol Vis Sci.

[54] Libby RT, Kitamoto J, Holme RH, Williams DS, Steel KP (2003). Cdh23 mutations in the mouse are associated with retinal dysfunction but not retinal degeneration.. Exp Eye Res.

[55] Libby RT, Steel KP (2001). Electroretinographic anomalies in mice with mutations in Myo7a, the gene involved in human Usher syndrome type 1B.. Invest Ophthalmol Vis Sci.

[56] Hemler ME (2008). Targeting of tetraspanin proteins–potential benefits and strategies.. Nat Rev Drug Discov.

[57] Reiners J, van Wijk E, Marker T, Zimmermann U, Jurgens K (2005). Scaffold protein harmonin (USH1C) provides molecular links between Usher syndrome type 1 and type 2.. Hum Mol Genet.

[58] Maerker T, van Wijk E, Overlack N, Kersten FF, McGee J (2008). A novel Usher protein network at the periciliary reloading point between molecular transport machineries in vertebrate photoreceptor cells.. Hum Mol Genet.

[59] Jacobson SG, Cideciyan AV, Aleman TS, Sumaroka A, Roman AJ (2008). Usher syndromes due to MYO7A, PCDH15, USH2A or GPR98 mutations share retinal disease mechanism.. Hum Mol Genet.

[60] Reiners J, Marker T, Jurgens K, Reidel B, Wolfrum U (2005). Photoreceptor expression of the Usher syndrome type 1 protein protocadherin 15 (USH1F) and its interaction with the scaffold protein harmonin (USH1C).. Mol Vis.

[61] Kitadokoro K, Bordo D, Galli G, Petracca R, Falugi F (2001). CD81 extracellular domain 3D structure: insight into the tetraspanin superfamily structural motifs.. EMBO J.

[62] Min G, Wang H, Sun TT, Kong XP (2006). Structural basis for tetraspanin functions as revealed by the cryo-EM structure of uroplakin complexes at 6-A resolution.. J Cell Biol.

[63] Lin HW, Schneider ME, Kachar B (2005). When size matters: the dynamic regulation of stereocilia lengths.. Curr Opin Cell Biol.

[64] Prosser HM, Rzadzinska AK, Steel KP, Bradley A (2008). Mosaic complementation demonstrates a regulatory role for myosin VIIa in actin dynamics of stereocilia.. Mol Cell Biol.

[65] Rhodes CR, Hertzano R, Fuchs H, Bell RE, de Angelis MH (2004). A Myo7a mutation cosegregates with stereocilia defects and low-frequency hearing impairment.. Mamm Genome.

[66] Holme RH, Kiernan BW, Brown SD, Steel KP (2002). Elongation of hair cell stereocilia is defective in the mouse mutant whirler.. J Comp Neurol.

[67] Angrand PO, Daigle N, van der Hoeven F, Scholer HR, Stewart AF (1999). Simplified generation of targeting constructs using ET recombination.. Nucleic Acids Res.

[68] Hayashi T, Cunningham D, Bermingham-McDonogh O (2007). Loss of Fgfr3 leads to excess hair cell development in the mouse organ of Corti.. Dev Dyn.

[69] Johnson PT, Brown MN, Pulliam BC, Anderson DH, Johnson LV (2005). Synaptic pathology, altered gene expression, and degeneration in photoreceptors impacted by drusen.. Invest Ophthalmol Vis Sci.

[70] Lee ES, Flannery JG (2007). Transport of truncated rhodopsin and its effects on rod function and degeneration.. Invest Ophthalmol Vis Sci.

[71] Hale IL, Matsumoto B (1993). Resolution of subcellular detail in thick tissue sections: immunohistochemical preparation and fluorescence confocal microscopy.. Methods Cell Biol.

[72] Geller SF, Ge PS, Visel M, Flannery JG (2008). In vitro analysis of promoter activity in Muller cells.. Mol Vis.

