# Supplementary material for: CLRN1 Is Nonessential in the Mouse Retina but Is Required for Cochlear Hair Cell Development
Source: PLoS Genet. 2009 Aug 14;5(8):e1000607. doi: 10.1371/journal.pgen.1000607 (PMC2719914; doi:10.1371/journal.pgen.1000607)
Supplement: Figure S1 — Oligonucleotide primers and fluorescent probes used. (0.40 MB PDF) [file pgen.1000607.s001.pdf]

**Primers**	**Figure #**	**Oligo Name**	**Oligo Sequence (5'-3')**
	1B, 1D, 5, 6	Clrn1-F2	TCATGCCAAGCCAGCAGAAGAAG
	1B, 1D, 5, 6	Clrn1-R2	CCTCCTGCTTCTGTTATTTTCC
	1B	Clrn1-R3	CCATACTGCATCTCGCCCATGAAC
	1B	Clrn1-R4	CTCTCCTTTGTCCTCATACAGAGAGTACC
	1B	Clrn1-R5	AGCCCCAGTGGTCCATGAAGAG
	1B, 4	Clrn1-R6	AAGGCGGTGGACTTTCACTTCAG
	1E-H, 4, 5	Clrn1-F1	GGTCCAAGCCATCCCCGTA
	1E-H, 4, 5	Clrn1-R1	TGTTCTGTAGGCATAGGTCCCTTC
	1C	Clrn1-F3	AGGCAATGTGGGTTAGGAGCAAG
	1C	Clrn1-R1	TGTTCTGTAGGCATAGGTCCCTTC
	1C	Act-F1	ACCAACTGGGACGACATGGAGAA
	1C	Act-R1	CATGGCTGGGGTGTTGAAGGT
	4, S2	28S-F1	GCGGTACACCTGTCAAAC
	4, S2	28S-R1	AGAGGCGTTCAGTCATAATC
	4	cFos-F1	CACCGACCTGCCTGCAAG
	4	cFos-R1	TCCAGCACCAGGTTAATTCCAATAATG
	4	Clrn1-F4	GCCATCCCCGTAAGCATCCAC
	4	Grm1-F1	GCCCTCTTCTCAGTCCATCACC
	4	Grm1-R1	ACTTCATCTCTGTCTGCCCATCC
	4	Rho-F1	CTACTTCGTCTTTGGGCCCACAG
	4	Rho-R1	CCCATAGCAGAAGAAGATGACGATCA
	4, 5, S2	Rp1-F1	ACGACATACAAAAGTACTTGCTTCCTGC
	4, 5, S2	Rp1-R1	GCAATCATATGTTTTCTCCGAGATCAG
	4	Slc1a3-F1	GGGAAGATGGGGATGCGAG
	4	Slc1a3-R1	GCCGAAGCACATGGAGAAG
	4	Thy1-F1	CGCGTCACCCTCTCCAACCA
	4	Thy1-R1	AACCAGCAGGCTTATGCCGC
	4	Vim-F1	GAATGACCGCTTTGCCAACTACAT
	4	Vim-R1	GCTTCCTCTCTCTGGAGCATCTCCT
	S2	Clrn1-F7 (rat)	CACAGAAGCCGTTTCTCATCATGC
	S2	Clrn1-R7 (rat)	ACCATGGTTAAGACGACAAGAATCATG
**Probes**	**Figure #**	**Probe Name**	**Probe Sequence (5'-FAM-3'-BHQ)**
	1C	Clrn1-1,3	TTCCGGTTCTCATTCTTCCCAGATTTGG
	1C	Clrn1-1,4	TTCCGGTTCTCATGCTCCTGTGG
	1C	Actb	CTGGCACCACACCTTCTACAATGAGC
