# Supplementary figures and images for: CLRN1 Is Nonessential in the Mouse Retina but Is Required for Cochlear Hair Cell Development
Source: PLoS Genet. 2009 Aug 14;5(8):e1000607. doi: 10.1371/journal.pgen.1000607 (PMC2719914; doi:10.1371/journal.pgen.1000607)

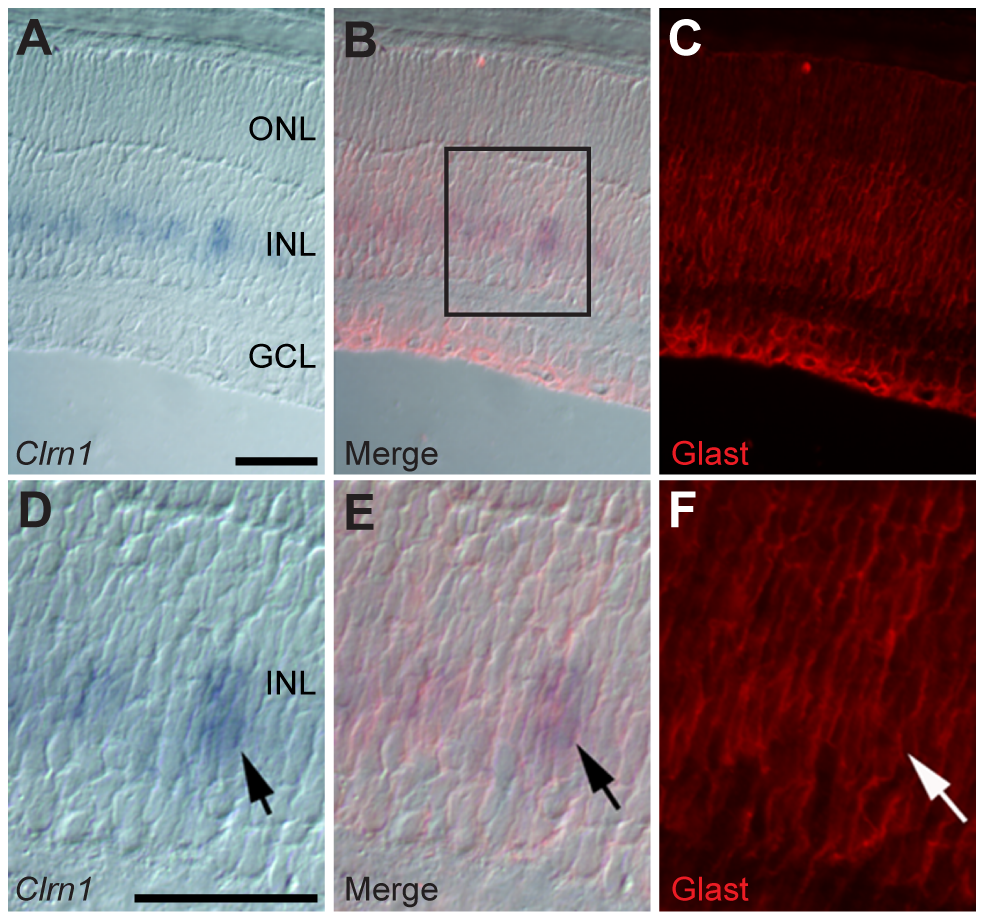

Supplement: Figure S2 — Clrn1 mRNA co-localizes with a marker for Müller cells. (A–C) Clrn1 ISH combined with anti-Glast (Slc1a3; a Müller glial marker) immunohistochemistry labeling shows co-labeling. The labeling in the GCL represents Müller cell processes, which extend to and surround cells in the GCL. (D–F) Higher magnification view of the region in the box in H, and the arrow points to a cluster of cells that is labeled with both Clrn1 and Glast. Scale bars = 50 µm. (1.42 MB TIF) [file pgen.1000607.s002.tif]

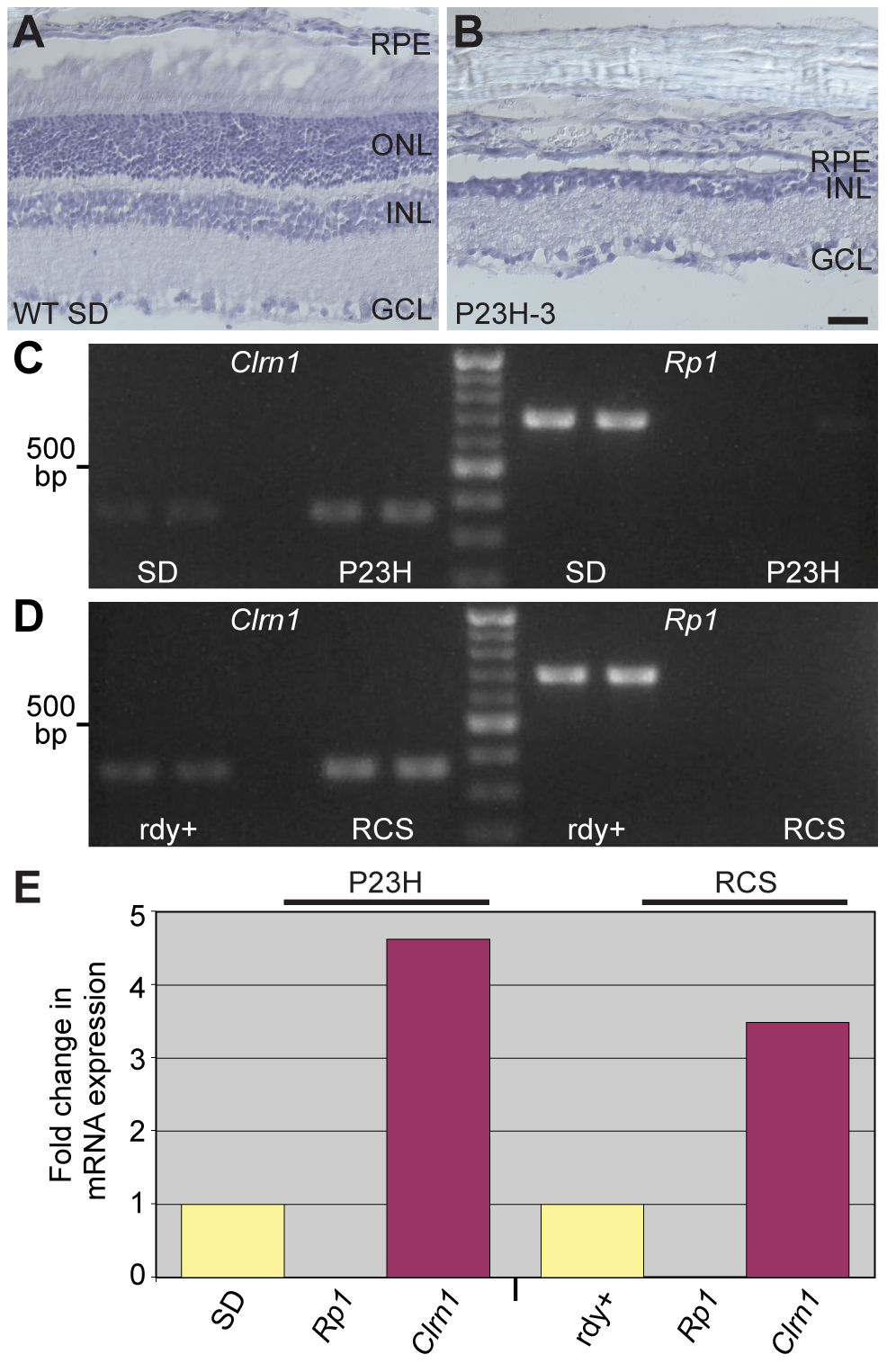

Supplement: Figure S3 — Genetically Degenerated rat retinas express Clrn1 despite photoreceptor loss. (A,B) Photomicrographs of retinal cross-sections from a two month old WT Sprague-Dawley rat (A) and a 1 year old P23H-3 (P23H-3; rhodopsin mutant) transgenic rat (B). Note the apposition of the RPE and INL in the P23H retina, and thus, a complete absence of the outer nuclear layer (ONL). Tissues were stained with hematoxylin. (C) Using RT-PCR, both SD and P23H rats (duplicate samples for each) express Clrn1. In contrast, only the WT animal expresses Rp1, supporting the histological loss of photoreceptors in the P23H animals. (D) Similar results are observed in the RCS rat. The congenic control strain (rdy+) expresses both Clrn1 and Rp1 by RT-PCR, whereas the RCS mutant animals express Clrn1, but not Rp1. (E) Quantitative RT-PCR analysis in the two rat models of photoreceptor degeneration indicates ∼100-fold reductions in the expression of Rp1. Due to equal amounts of retinal cDNA being used in each reaction, a relative increase in Clrn1 expression is observed in mutant animals due to the loss of photoreceptor mRNA in the total retinal RNA pool used for cDNA synthesis. Data represent the averages of two animals in each condition. Scale bar = 50 µm. (1.04 MB TIF) [file pgen.1000607.s003.tif]

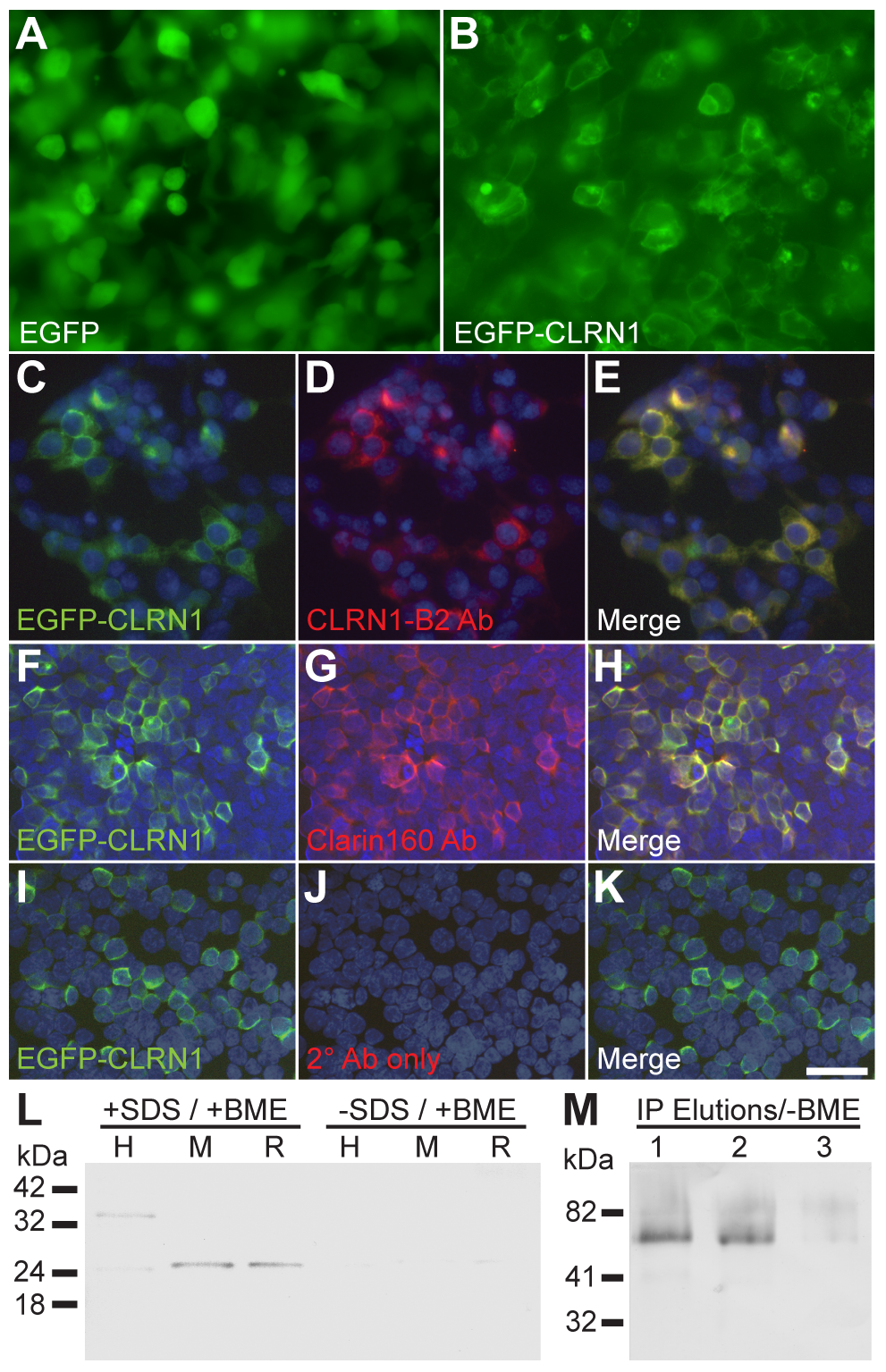

Supplement: Figure S4 — Clarin 1 localizes to the plasma membrane in transiently transfected HEK293 cells, and anti-CLRN1 antibodies specifically detect CLRN1. Clarin 1 Localizes to the Plasma Membrane in Transiently Transfected HEK293 Cells, and Anti-CLRN1 Antibodies Specifically Detect CLRN1. (A) Transfection with the pEGFP-C1 parent plasmid alone results in bright green cytoplasmic fluorescence in HEK293 cells. (B) The mouse Clrn1 cDNA (exons 1, 3, and 4) was cloned in-frame with EGFP. Following transfection, the EGFP-CLRN1 fusion protein caused the redistribution of EGFP (green fluorescence) from the cytoplasm to the plasma membrane. (C–E) CLRN1-B2 antibody staining of HEK293 cells transfected with the EGFP-CLRN1 fusion protein. The merged image (E) shows near perfect co-localization (yellow). Cells were counterstained with DAPI. (F–H) HEK293 cells were transfected with the EGFP-CLRN1 fusion protein construct and immunostained with a different anti-CLRN1 antibody (Clarin160). (I–K) Cells were transfected with the EGFP-CLRN1 fusion protein construct and immunostained with secondary antibodies alone. Only green fluorescence originating from EGFP is visible. (L) Western blot using the CLRN1-B2 antibody on human (H), mouse (M), and rat (R) retinal homogenates, with and without SDS in the sample buffer, including BME. The CLRN1-B2 antibody detects a ∼26 kDa band in mouse and rat, and a doublet of ∼25 kDa and ∼34 kDa in the human retinal sample. (2.01 MB TIF) [file pgen.1000607.s004.tif]

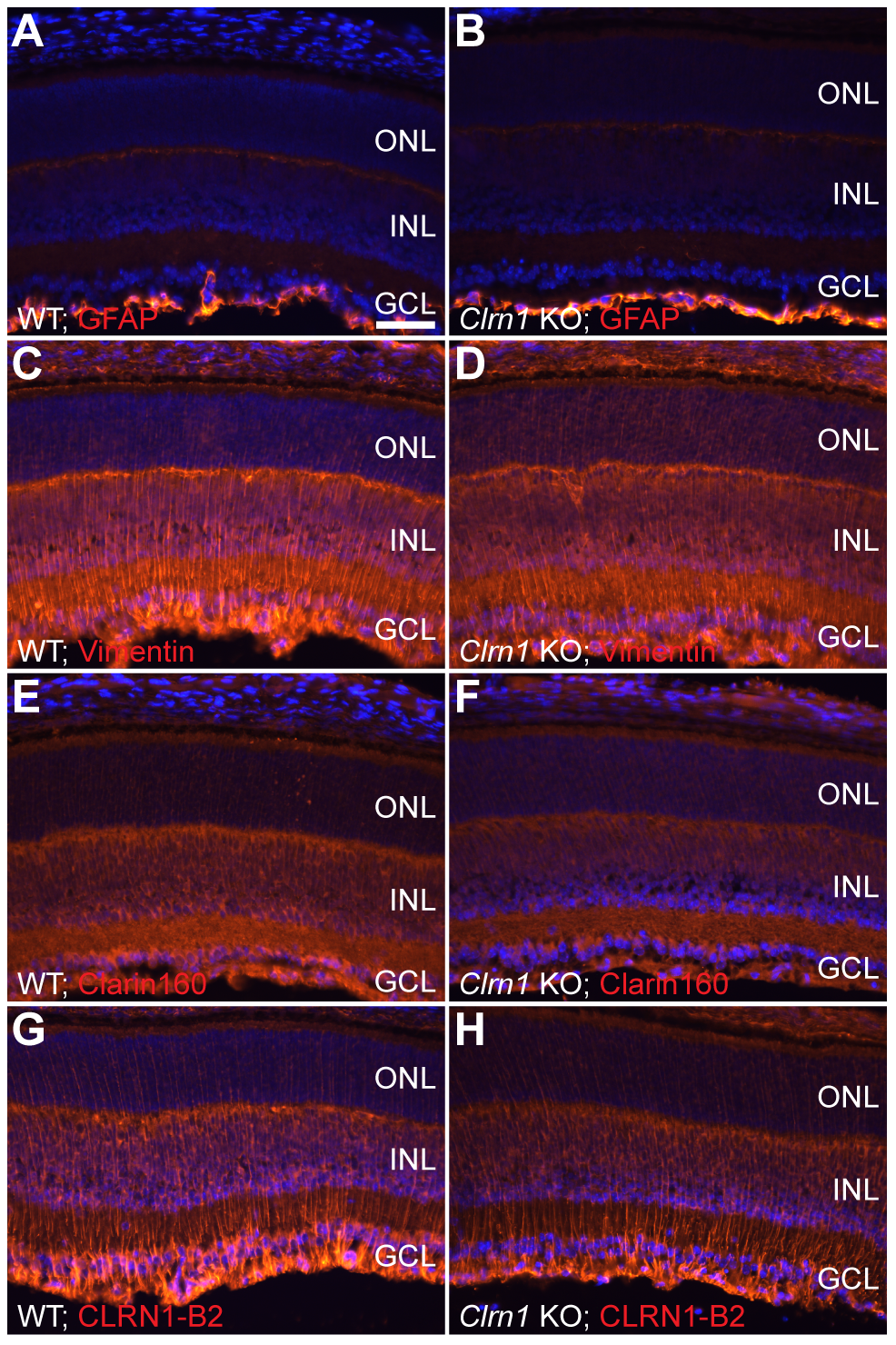

Supplement: Figure S5 — Immunostaining of P7 WT and Clrn1 KO mouse retinal sections. (A–H) Similar staining patterns were observed in WT and Clrn1 KO P7 retinal tissues immunostained with GFAP (A–B), vimentin (C–D), Clarin160 (E–F), and CLRN1-B2 antibodies. Though there may be a slight reduction in labeling in the Clrn1 KO mouse (F and H), non-specific (background) staining in the tissues prevents reliable use of either anti-CLRN1 antibody to specifically localize CLRN1 in the retina. Scale bar = 20 µm. ONL = outer nuclear layer, INL = inner nuclear layer, and GCL = ganglion cell layer. (2.74 MB TIF) [file pgen.1000607.s005.tif]

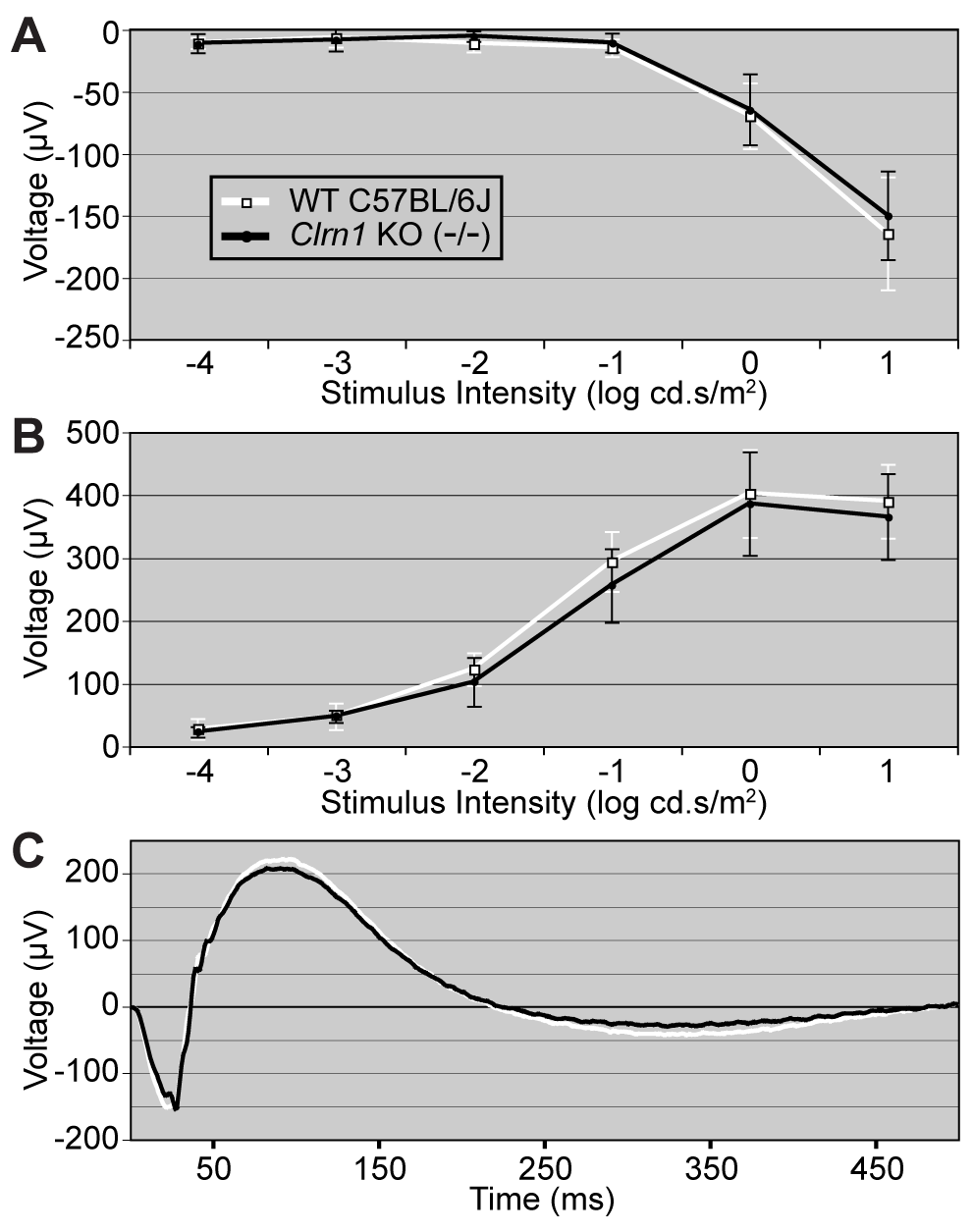

Supplement: Figure S6 — Electroretinograms of WT and Clrn1 KO (−/−) mice at 18 months of Age. (A,B) White traces represent WT C57BL/6J mice, while black traces represent Clrn1 KO (−/−) mice. (A) Peak A-wave voltage (µV) responses to increasing light intensities (log cd.s/m2), from left to right. (B) Peak B-wave voltage responses to increasing light intensities, from left to right. Both A- and B- waves are similar for the WT and Clrn1 KO (−/−) animals. (C) Graph illustrating voltage changes vs. time for both the WT and Clrn1 KO (−/−) animals. Error bars = 1 SD. (0.13 MB TIF) [file pgen.1000607.s006.tif]

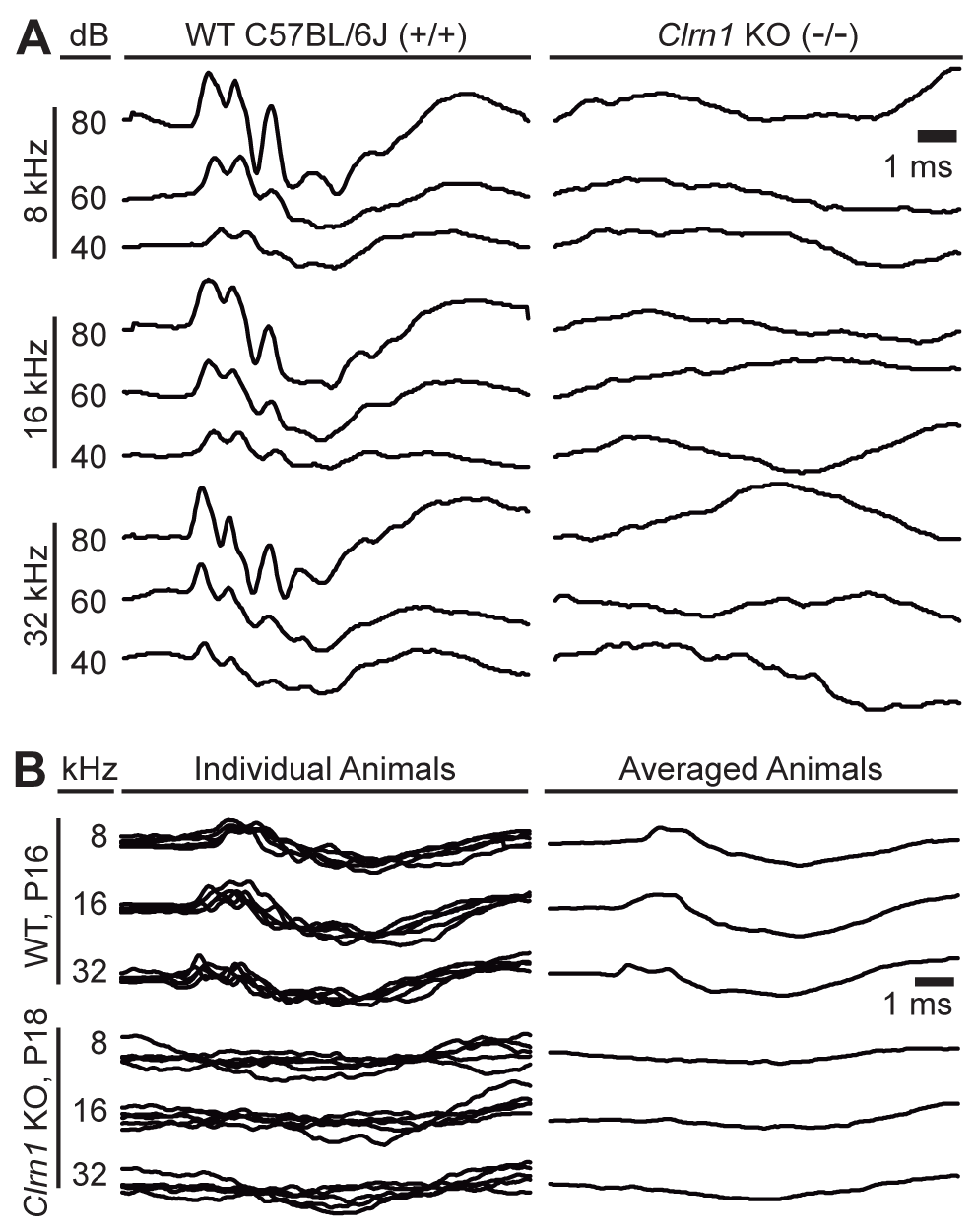

Supplement: Figure S7 — Auditory brainstem response recordings of inner ear function. (A) Ten millisecond (ms) auditory brainstem responses (ABR) in WT C57BL/6J and Clrn1 KO (−/−) mice at 6 weeks of age. Averaged traces are shown for five animals in each condition, exposed to 3 sound intensities (40, 60, and 80 dB SPL) at three frequencies (8, 16, and 32 kHz). Clrn1 KO animals exhibit a profound diminution in their brainstem responses at all the dB SPL levels tested, and tonal frequency had no effect on the ABR. (B) Ten ms 80 dB SPL Auditory Brainstem Response (ABR) recordings from WT C57BL/6J (P16) and Clrn1 KO (P18) mice. Five animals were recorded for each genotype; individual traces are shown on the left, and averaged curves are shown on the right. Even at this early age, Clrn1 KO animals show severely depressed ABR sensitivities. A 1 ms bar is shown for reference. (0.17 MB TIF) [file pgen.1000607.s007.tif]

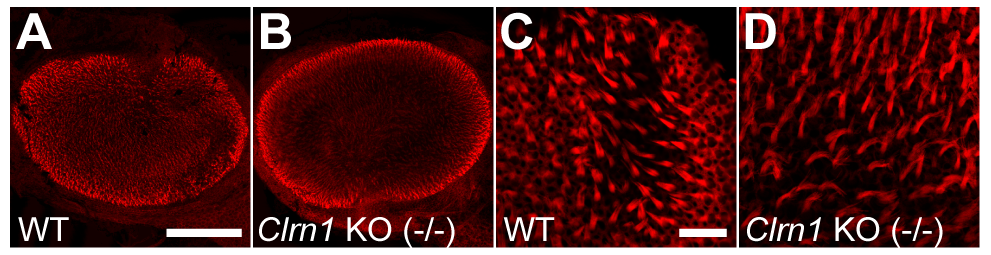

Supplement: Figure S8 — P120 vestibular hair cells of Clrn1 KO mice appear unaffected. (A–D) Immunofluorescent confocal images of inner ear sensory maculae harvested from utricles. Samples were labeled with rhodamine-Phalloidin and are shown at low (A and B) and high (C and D) magnification. Clrn1 KO mice show a grossly normal utricular elliptical shape (B) with classical distribution of hair cells (D). Scale bars: In A (200 µm) applies to A and B; in C (20 µm) applies to C and D. (0.33 MB TIF) [file pgen.1000607.s008.tif]
